# Peanut-Shaped Gold Nanoparticles with Shells of Ceragenin CSA-131 Display the Ability to Inhibit Ovarian Cancer Growth In Vitro and in a Tumor Xenograft Model

**DOI:** 10.3390/cancers13215424

**Published:** 2021-10-29

**Authors:** Ewelina Piktel, Ilona Oscilowska, Łukasz Suprewicz, Joanna Depciuch, Natalia Marcińczyk, Ewa Chabielska, Przemysław Wolak, Katarzyna Głuszek, Justyna Klimek, Piotr M. Zieliński, Michał T. Marzec, Paul B. Savage, Magdalena Parlińska-Wojtan, Robert Bucki

**Affiliations:** 1 Department of Medical Microbiology and Nanobiomedical Engineering, Medical University of Białystok, 15-222 Białystok, Poland; ewelina.piktel@wp.pl (E.P.); lukaszsuprewicz@gmail.com (Ł.S.); 2 Department of Analysis and Bioanalysis of Medicines, Medical University of Białystok, 15-089 Białystok, Poland; ilona.zareba@gmail.com; 3 Institute of Nuclear Physic, Polish Academy of Sciences, Radzikowskiego 152, 31-342 Krakow, Poland; joannadepciuch@gmail.com (J.D.); pm.zielinski@ifj.edu.pl (P.M.Z.); magdalena.parlinska@ifj.edu.pl (M.P.-W.); 4 Department of Biopharmacy, Medical University of Białystok, 15-222 Białystok, Poland; natalia.marcinczyk@umb.edu.pl (N.M.); ewa.chabielska@umb.edu.pl (E.C.); 5 Institute of Medical Science, Collegium Medicum, Jan Kochanowski University of Kielce, 25-001 Kielce, Poland; przemyslaw.wolak@ujk.edu.pl (P.W.); kasia.kielce@wp.pl (K.G.); 6 Department of Human Anatomy, Medical University of Bialystok, 15-230 Białystok, Poland; justyna.klimek@umb.edu.pl; 7 Laboratory of Immuno-Endocrinology, Inflammation, Metabolism, and Oxidation Section, Department of Biomedical Sciences, University of Copenhagen, DK-2200 Copenhagen, Denmark; michal@sund.ku.dk; 8 Department of Chemistry and Biochemistry, Brigham Young University, Provo, UT 84604, USA; pbsavage@chem.byu.edu

**Keywords:** nanotechnology, drug delivery, non-spherical gold nanoparticles, gold nanopeanuts, ovarian cancer, ceragenins

## Abstract

**Simple Summary:**

Despite a spectrum of therapeutics available for the treatment of ovarian tumors, there is a constant need to develop novel treatment options, particularly due to a high incidence of drug resistant tumors and low 5-year survival of patients diagnosed with ovarian carcinomas. In this study, we employed a nanotechnology-based approach to present a novel nanosystem based on ceragenin CSA-131 attached to the surface of a peanut-shaped gold nanoparticle. We demonstrate that such a prepared nanoformulation was highly effective against ovarian cancer cells in in vitro settings and, with limited toxicity, was able to prevent the growth of ovarian tumors in treated animals. Based on obtained data we suggest that ceragenin-containing nanosystems should be considered and further tested as potential therapeutics for ovarian malignancy.

**Abstract:**

Gold nanoparticles-assisted delivery of antineoplastics into cancerous cells is presented as an effective approach for overcoming the limitations of systemic chemotherapy. Although ceragenins show great potential as anti-cancer agents, in some tumors, effective inhibition of cancer cells proliferation requires application of ceragenins at doses within their hemolytic range. For the purpose of toxicity/efficiency ratio control, peanut-shaped gold nanoparticles (AuP NPs) were functionalized with a shell of ceragenin CSA-131 and the cytotoxicity of AuP@CSA-131 against ovarian cancer SKOV-3 cells and were then analyzed. In vivo efficiency of intravenously and intratumorally administered CSA-131 and AuP@CSA-131 was examined using a xenograft ovarian cancer model. Serum parameters were estimated using ELISA methods. Comparative analysis revealed that AuP@CSA-131 exerted stronger anti-cancer effects than free ceragenin, which was determined by enhanced ability to induce caspase-dependent apoptosis and autophagy processes via reactive oxygen species (ROS)-mediated pathways. In an animal study, AuP@CSA-131 was characterized by delayed clearance and prolonged blood circulation when compared with free ceragenin, as well as enhanced anti-tumor efficiency, particularly when applied intratumorally. Administration of CSA-131 and AuP@CSA-131 prevented the inflammatory response associated with cancer development. These results present the possibility of employing non-spherical gold nanoparticles as an effective nanoplatform for the delivery of antineoplastics for the treatment of ovarian malignancy.

## 1. Introduction

Ovarian cancer originating in cells on the outer surface of the ovary or in the fallopian tube epithelium is recognized as the most fatal of all gynecological tumors. Statistically, 21,410 women will be diagnosed with ovarian cancer in 2021 in the United States alone and about 13,770 women will die [1]. Non-specific symptoms of ovarian cancer as well as a lack of universal detection methods are responsible for the high staging of the disease in 70% of patients at the beginning of treatment, which makes their prognosis for survival very poor [2]. Conventional therapy of ovarian carcinoma includes neoadjuvant chemotherapy with subsequent surgical intervention [3]. Considering the limitations of systemic chemotherapy that result from the significant toxicity of the antineoplastic drugs associated with the administration of the high doses that are required to achieve an appropriate concentration in the target tumor, as well as the development of an accompanied drug resistance, new methods to limit cancer drug toxicity are urgently needed [4]. In particular, cancer cell drug resistance is largely responsible for the recurrence of the disease that is observed in up to 60–80% of patients [5]. One approach that can lead to an improvement in the antitumor effect of drugs is the development of carriers that allow the therapeutic agent to be delivered in an effective concentration to diseased sites without affecting normal cells [4,6]. Particularly, multifunctional materials, whose unique physicochemical properties facilitate their employment for simultaneous drug delivery and imaging of therapy effectiveness, are of the greatest interest.

Gold nanoparticles (Au NPs), due to their set of physicochemical, optical and electronic properties, have gained considerable interest, particularly with regard to their utility as advanced systems for cancer imaging and treatment [7]. Extensive surface-to-volume ratio and relatively good biocompatibility, as well as highly optimized methods for Au NPs synthesis that allow for modification of their morphological features and surface chemistries, thus facilitating effortless decoration with a spectrum of diagnostic and therapeutic agents, makes gold nanoparticles highly feasible materials for use in the design of drug delivery vehicles [8]. Accordingly, gold nanoparticles were presented as being effective for targeted drug delivery into ovarian cancer cells and overcoming chemoresistance [9], restoring impaired gene function [10] or for co-administering two antineoplastic agents at the same time [11]. Importantly, due to highly optimized synthesis protocols, gold nanoparticles might be effectively tailored by developing nanostructures with adjustable sizes and shapes, which inherently determine their bioactivity in vivo and their intracellular fate [12,13]. When compared with spherical Au NPs, which are most widely used in drug delivery applications [14], gold nanoparticles with modified morphology are mostly recognized as those with an enhanced drug loading surface [15], improved biodistribution [16], higher cytotoxicity and higher cellular uptake efficiency [17], although some reports are equivocal in this manner [17,18,19]. This points out that the desired biological activity of metallic nanomaterials and their in vivo behavior is a result of such features as size, shape or surface chemistry [20]. Some studies also suggest that non-spherical gold nanomaterials might be less biocompatible than spherical ones [21], which surely calls for some caution in their in vivo application. Certainly, however, non-spherical metallic nanoparticles, due to a spectrum of valuable features, are interesting candidates for use as components of upgraded anti-cancer nanoformulations.

As a continuation of our previous in vitro study demonstrating the potent anti-cancer activity of peanut-shaped gold nanoparticles (AuP NPs) when compared with spherical-shaped nanomaterials ([22]; see Supplementary Materials), we examined the usefulness of these elongated-type nanostructures as carriers for the delivery of ceragenin CSA-131 into ovarian cancer cells. Ceragenins, due to their favorable pharmacokinetic and pharmacodynamic features, such as non-specific potent membrane activity, stability in physiological fluids and reduced susceptibility to development of drug resistance, have been successfully tested as potent antimicrobials with clinical applicability for the treatment of drug-resistant bacteria and fungal infections [23,24,25,26]. In addition, a growing body of evidence highlights the great potential of ceragenins as a novel anti-cancer therapeutic, although the research that has been demonstrated to date was carried out only in in vitro settings and was never confirmed in animal models [27]. Wide-ranging research using cancer cell lines with the greatest clinical implications indicates that ceragenins CSA-13 and CSA-131 exert anti-cancer activity in colon cancer [28,29], breast cancer [30] and lung adenocarcinoma [27]. Investigation of the mechanism of action has shown that ceragenins regulate cancer development via disruption of redox stability in treated cancer cells and induction of both caspase-independent and caspase-dependent apoptosis [29,30]. An important limitation, however, hindering the use of ceragenins as effective anti-cancer agents for systemic administration is their hemolytic activity observed in the dose range, which is typically required for cancer growth inhibition [31]. For this reason, improved ways of reducing the effective anti-cancer doses of ceragenin without increasing their toxicity is particularly desirable. It has been proposed that using gold nanoparticles to deliver the drug may be applicable in this regard.

The purpose of this work was to elucidate the therapeutic efficiency of peanut-shaped gold nanoparticles functionalized by ceragenin CSA-131 (AuP@CSA-131) against ovarian cancer. The study was performed using both (i) an in vitro model employing the SKOV-3 ovarian cancer cell line and (ii) in a xenograft in vivo experimental setting, where compounds were administered intravenously (i.v.) or intratumorally (i.t.). Two questions were asked: does combining of CSA-131 with gold nanopeanuts into one nanosystem allow lowering the effective doses of CSA-131 to a concentration that might be considered non-toxic, and what mechanism of action determines the killing activity of such a nanoformulation? According to our best knowledge, this is the first study investigating the effectiveness of free ceragenins and ceragenin-containing nanosystems in a xenograft model of cancer.

## 2. Material and Methods

### 2.1. Synthesis of AuP@CSA-131 Nanosystem and Its Labelling by IRDye^®^ 800CW

In the first step of AuP@CSA-131 synthesis, water-based solutions of CTAB (364 mg in 5 mL of H_2_O), HAuCl_4_ (1.7 mg in 10 mL of H_2_O) and NaBH_4_ (0.1 M; 0.6 mL) were prepared and added into the beaker. The reaction was performed under vigorous stirring and stopped when the solution turned red (solution A). In the second step of synthesis, 5 mL of solution B (364 mg of CTAB in 5 mL of H_2_O), 0.2 mL of solution C (13.5 mg AgNO_3_ in 20 mL of H_2_O), 5 mL of solution D (1.7 mg HAuCl_4_ in 5 mL of H_2_O_2_), 70 μL of solution E (138.6 mg C_6_H_8_O_6_ in 10 mL of H_2_O) and 30 μL of solution A were poured into a beaker. The reaction was continued for 30 min, and the obtained AuP NPs were incubated overnight with MHDA at 4 °C. MHDA was used in a considerable excess to ensure that all ceragenin molecules added in the subsequent steps would attach to the surface of the AuP NPs. Next, the solution was rinsed with dimethylformamide (DMF), and the MHDA-functionalized gold nanostructures were maintained in DMF solution of diisopropylethylamine (DIPEA), pentafluorophenyl (PFP) and *N*-cyclohexyl-*N*′-(2-morpholinoethyl) carbodiimide metho-p-toluenesulfonate (CMC) for 30 min at 25 °C. The prepared nanoformulation was repeatedly rinsed and centrifuged before the addition of CSA-131 for 30 min at 25 °C. The amounts of CSA-131 and AuP NPs in the final nanosystem were 2 mg/mL (2 mM, i.e., 1.204 × 10^21^ molecules) and 2.93 ng/mL (1.5 × 10^−8^ M, i.e., 9.03 × 10^15^ molecules), respectively. Based on this, ceragenin loading was calculated as 1.33 × 10^5^ CSA-131 molecules per nanoparticle.

For the purpose of AuP@CSA-131 biodistribution analysis in the animal model, the nanosystem was labelled with IRDye^®^ 800CW by mixing AuP@CSA-131 and IRDye^®^ 800CW NHS ester in a 10:1 ratio. The reaction between free amino groups from CSA-131 and the *N*-hydroxysuccinimide (NHS) reactive group of IRDye^®^ 800CW was continued overnight at 4 °C.

### 2.2. Physicochemical Properties of Gold Nanopeanuts and Verification of Success of Functionalization and CSA-131 Immobilization Process

Physicochemical characterization of gold nanopeanuts, as well of the ceragenin-coated products, was performed using (i) high-angle annular dark-field scanning transmission electron microscopy (HAADF-STEM), (ii) Fourier Transform Raman (FT-Raman) and (iii) thermogravimetric (TGA) analysis. The morphology of the developed nanomaterials was explored by analyzing of 100 NPs from HRSTEM images [18]. Fourier Transform Raman (FT-Raman) spectra were collected to determine the extent and stability of nanoparticles functionalization, CSA-131 immobilization and labelling by IRDye^®^ 800CW processes, using the same parameters as in our previous paper [32]. For the purpose of exploring the thermal stability of both unlabeled AuP@CSA-131 and nanomaterials marked with IRDye^®^ 800CW, thermogravimetric (TGA) analysis was conducted using the following parameters: temperature up to 500 °C; heating rate: 5 and 10 °C/min. Nickel and alumel standards were used to perform the temperature calibration. An indium standard was used to perform calibrations of temperature and enthalpy.

### 2.3. Cell Culture

Human ovarian adenocarcinoma cells SKOV-3 (ATCC^®^ HTB-77™) were cultured in McCoy’s 5A complemented with 10% fetal bovine serum (FBS), 2 mM/L glutamine and 1% of antibiotics (penicillin, streptomycin, amphotericin B) at 37 °C (5% CO_2_). For experiments, cells were seeded at a density of 10,000 or 50,000 cells/well (for 96-well plates and 24-well plates, respectively) and cultured with CSA-131 or AuP@CSA-131 at doses of 2, 5 and 10 µg/mL for 24, 48 and 72 h (for cytotoxicity evaluation) or 72 h exclusively (for mechanism determination assays).

### 2.4. Cytotoxicity Measurements

In the initial step of the study, differences in cytotoxic activity of CSA-131 and AuP@CSA-131 were estimated using MTT assay, as described in the previous reports [30,33] and demonstrated as the viability percentage when compared with the control cells (0 µg/mL, 100% of survival). By extrapolation from each dose–response curve, doses required to decrease the cellular viability by 50%, 75% and 90% (IC50, IC75 and IC90, respectively) were calculated, and relative inhibitory effects were estimated (Table 1). Alterations in morphological features of CSA-131 and AuP@CSA-131-treated cells were investigated using light microscopy at 40× magnification.

### 2.5. Immunofluorescence Staining of Apoptosis and Autophagy-Related Proteins

Immunostaining of cells exposed to CSA-131 and AuP@CSA-131 for 72 h was performed in order to visualize alterations in the expression of proteins related to cell killing processes. For this purpose, treated SKOV-3 cells were fixed, permeabilized, blocked with 0.1% bovine serum albumin (BSA) and incubated with antibodies against apoptotic (NOX4, annexin A1, cleaved caspase-3, cleaved caspase-9) and autophagic proteins (Atg12 and Beclin-1) according to the previously presented protocol [22].

### 2.6. Western Blot Analyses

To quantitively estimate the alterations in ceragenin-affected signaling pathways, SKOV-3 cells were cultured for 72 h with increasing concentrations of CSA-131 and AuP@CSA-131, followed by Western blot analysis, according to the protocol in our previous paper [22].

### 2.7. Intracellular GSH Level Estimation

VitaBright-48^™^ (VB-48^™^)-based assay designated for NucleoCounter^®^ NC-3000™ Cell Analyzer (Chemometek, Allerod, Denmark) was employed as a fluorescent method of intracellular GSH quantification. For this purpose, SKOV-3 cells were exposed to CSA-131 and AuP@CSA-131 at concentrations of 2–10 µg/mL for 72 h, washed twice with PBS, harvested and incubated with dye mixture containing VB-48^™^, acridine orange (AO) and propidium iodide (PI). AO/PI dual-staining was used to discriminate fractions of (i) healthy cells, (ii) PI-negative cells with low viability and (iii) dead cells from the treated cellular population.

### 2.8. ROS Production Investigation

Intracellular reactive oxygen species formation was measured using 2′-7′-dichlorofluorescin diacetate (DCFH-DA) at a final concentration of 20 µM. Cells pre-incubated with DCFH-DA SKOV-3 were treated with the tested compounds at indicated doses, and a ROS-derived fluorescence signal was recorded every 24 h (488/535 nm).

### 2.9. Mitochondrial Transmembrane Potential Analysis

Alterations in the functioning of ovarian cancer cells mitochondria upon exposure to CSA-131 and AuP@CSA-131 at doses of 2, 5 and 10 µg/mL were measured using the NucleoCounter^®^ NC-3000™ fluorescence image cytometer, according to the protocol presented in our previous paper [22].

### 2.10. Ovarian Cancer Xenograft Model

Athymic (Rj:ATHYM-foxn1nu/nu, 10-week-old, weighting 20–22 g) female, nude mice (*n* = 72) were obtained from Janvier Labs (Le Genest-Saint-Isle, France) and housed in autoclaved, individually ventilated cages (IVC cages). During the experiment, animals were maintained in a laboratory animal facility (19–23 °C, 50 ± 10% relative humidity, 12 h light/dark cycle) with unlimited access to sterile water and feed. Before the beginning of the study, animals were quarantined and acclimatized for 2 weeks. During the acclimatization period, no abnormalities in the health of the animals were observed; therefore, all animals were classified to continue the experiment. All animal experiments were performed in accordance with the guidelines of the Local Ethics Committee at the University of Warmia and Mazury in Olsztyn, Poland (no. 61/2019). Xenografts were established from the SKOV-3 cell line by the subcutaneous implantation of 1 × 10^6^ cells in 50 µL PBS into the flank of 48 mice. For animals that were considered as controls (*n* = 24 in total), 50 µL of sterile saline was injected subcutaneously. The appearance of a tumor and its volume (calculated as v = 0.5 × a × b^2^, where a and b are the largest and smallest tumor diameters measured using a caliper) were monitored up to day 10, when the average volume of tumors was approximately 160 mm^3^.

### 2.11. Treatment of Animals

On day 10, tumor-bearing mice (*n* = 48) were randomly divided into 8 groups (3 groups for biodistribution analysis and 6 groups for anti-cancer effectiveness investigation) with 3–6 mice per group. Five additional groups (*n* = 24 in total) were used as healthy controls for biodistribution testing (*n* = 18) and therapeutic efficiency assessment (groups containing from 3 to 6 animals). For biodistribution analysis, both healthy and cancer-bearing mice were treated intravenously with 10 µg/mL (1 mg/kg) of IRDye^®^ 800CW-labelled CSA-131 (CSA-131 (800CW)), AuP@CSA-131 (AuP@CSA-131 (800CW)) or unmodified IRDye^®^ 800CW, and at indicated time points after i.v. injection, mice were anesthetized and scanned at 800 nm for the targeted IRDye^®^ 800CW fluorescence signal [34]. For the purpose of anti-cancer effectiveness investigation, mice were treated intravenously or intratumorally every 72 h using CSA-131 or AuP@CSA-131 at a dose of 10 µg/mL (1 mg/kg) up to day 28 of the experiment (i.e., 7 doses of compounds administered in total). Finally, animals were sacrificed by induction of inhalation anesthesia with 4% isoflurane in medical oxygen and total heart bleeding. The collected blood was centrifuged, and plasma was frozen at −80 °C. A full postmortem examination was performed, including external visual inspection and internal organ evaluation. The following organs were collected from all animals for further examination: lung, kidney, spleen, heart, gastric system, pancreas, liver and cancerous tumor. The harvested organs were weighed and then fixed in a 10% buffered formalin solution. Fixed organs were stored for 72 h at room temperature and then placed in a refrigerator at 4 °C.

### 2.12. Investigation of Procalcitonine (PCT), Lactate Dehydrogenase (LDH) and Interleukin-6 (IL-6) Concentrations in Serum of Sacrificed Animals

PCT and LDH concentrations in the serum of the sacrificed animals were measured using commercially available colorimetric kits (Mouse Procalcitonin ELISA Kit, Novus Biologicals, Centennial, CO, USA and Cytotoxicity Detection Kit^PLUS^, Sigma-Aldrich, Saint Louis, MO, USA). Interlukin-6 (IL-6) serum concentrations were estimated using Mouse IL-6 Quantikine ELISA Kit (R&D Systems, Minneapolis, MN, USA).

### 2.13. Profiling of Inflammatory Factors Expression

Membrane-type antibody-pair-based array (Mouse Inflammation Antibody Array, abcam, Cambridge, UK) was used to investigate relative alterations of inflammation-associated factors among treated groups from pooled serum samples, respectively.

### 2.14. Statistical Analysis

Results of experiments are presented as mean ± SD. Statistical analysis was performed using the two-tailed Student’s *t*-test with OriginPro 2020 (OriginLab Corporation, Northampton, MA, USA). A value of *p* < 0.05 was considered to be statistically significant.

## 3. Results

### 3.1. Synthesis and Physicochemical Properties of AuP@CSA-131

**Figure 1:**
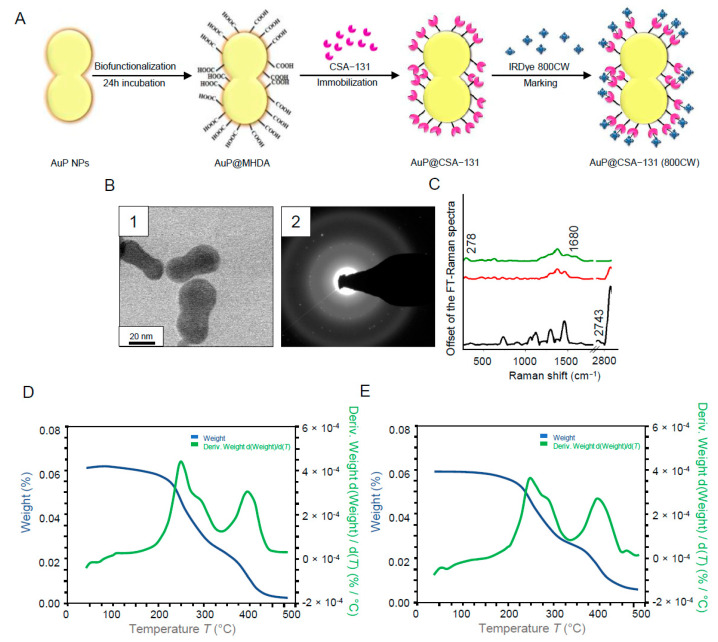
Schematic representation of the AuP NPs biofunctionalization, ceragenin (CSA) immobilization and labelling of AuP@CSA-131 with IRDye^®^ 800CW (**A**). STEM image of the obtained AuP NPs (**B1**) and SEAD patterns of gold nanoparticles used for nanosystem synthesis (**B2**). Unenhanced FT-Raman spectra of MHDA (black spectrum), CSA-131 immobilized on the AuP NPs surface (red spectrum) and CSA-131 immobilized on the AuP NPs surface marked with IRDye^®^ 800CW (green spectrum) (**C**). TGA data of AuP@CSA-131 (**D**) and AuP@CSA-131 (800CW) (**E**) showing the solvent removal and decomposition of the product.

The formation of the AuP@CSA-131 nanosystem consisted of the following five steps: (i) synthesis of gold nanoseeds, (ii) synthesis of peanut-shaped gold nanoparticles, (iii) biofunctionalization of AuP NPs with 16–mercaptohexadecanoic acid (MHDA) linker via -SH group of MHDA, (iv) attachment of ceragenin CSA-131 to the surface of the obtained product by forming a bond between the -COOH group of MHDA and -NH_2_ of ceragenin and (v) labelling with IRDye^®^ 800CW probe (Figure 1A). As demonstrated in Figure 1B, the developed gold nanostructures are characterized by a peanut-like shape with rounded edges. By measurement of 100 randomly selected nanoparticles, it was calculated that the size of the obtained NPs along the longitudinal axis was around 60 ± 5 nm, while along the transverse axis the size was 30 ± 3.5 nm, and the size spread of the Au NPs was recognized as negligible (Figure 1(B1)). Synthetized nanoparticles were classified as crystalline, as circles indexed with planes corresponding to the face-centered cubic (fcc) structure of Au were visible in SAED patterns (Figure 1(B2)) [35]. The correctness of the functionalization of AuP NPs with CSA-131 and IRDye^®^ 800CW was investigated using FT-Raman spectroscopy analysis (Figure 1C). Accordingly, the peak at 2743 cm^−1^ corresponding to the –SH group was recorded to be present in the FT-Raman spectrum of MHDA (black spectrum). These peak disappeared in the spectra of CSA-131 immobilized on the AuP NPs surface (red spectrum) and CSA-131 immobilized on the AuP NPs surface marked with IRDye^®^ 800CW (green spectrum). Moreover, in these two spectra, a peak at 278 cm^−1^ originating from Au-S stretching vibrations was visible. These two bonds were responsible for creating a connection between the surface of the gold nanopeanuts and the sulfur from MHDA [36]. Moreover, in the FT-Raman spectrum of AuP@CSA-131 (red spectrum), a Raman shift at 1680 cm^−1^ corresponding to the N-H vibrations was observed [37]. These groups are responsible for linking ceragenin with the biofunctional surfactants on the NPs’ surface [38]. Furthermore, as was recorded for samples consisting of IRDye^®^ 800CW-labelled CSA-131 attached to the surface of AuP NPs, the intensity of N-H vibrations was higher when compared with unlabeled AuP@CSA-131 (green spectrum). This is because the connection between IRDye^®^ 800CW led to the formation of a N-H bond [39]. The results of TGA and DSC experiments indicate that both the AuP@CSA-131 nanosystem and AuP@CSA-131 (800CW) remained thermally stable at high temperatures. The shape of the TGA signal pointed towards a strong evaporation of the solvent from the liquid sample visible between ca. 107 °C and 145 °C. At a temperature up to 150 °C, no signs of any significant thermal anomalies were observed in DSC thermogram. The baseline shift, as well as anomalies registered above that temperature, were effects of rapid boiling and decomposition of the sample or destabilization of MHDA (Figure 1D,E).

### 3.2. Immobilization of Ceragenin CSA-131 on the Surface of Peanut-Shaped Gold Nanoparticles Improves Its Anti-Cancer Activity against Ovarian Adenocarcinoma

**Figure 2:**
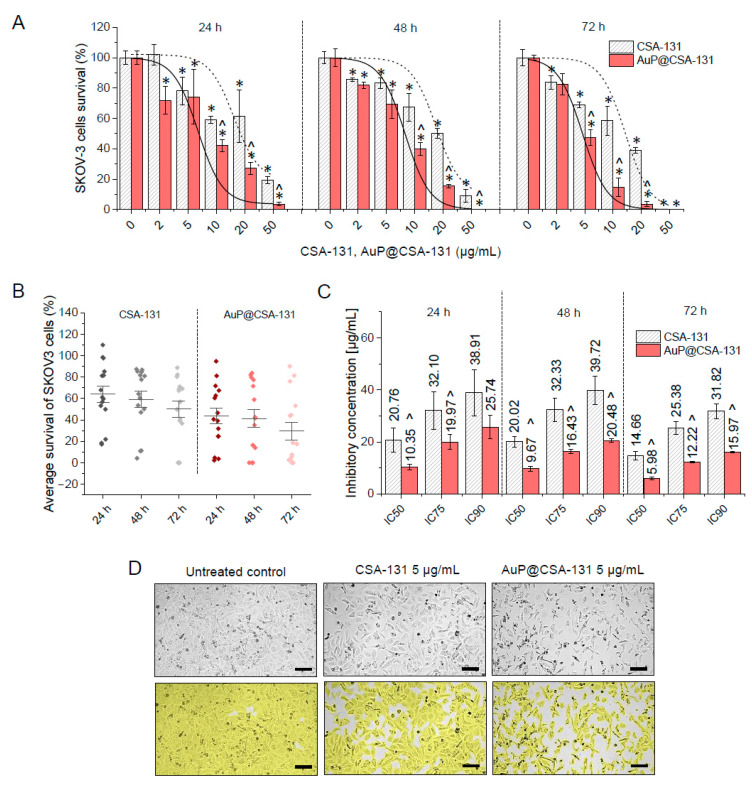
Cytotoxicity of ceragenin CSA-131 in free-form and as bound to peanut-shaped gold nanoparticles against ovarian cancer SKOV-3 cells. Decrease in SKOV-3 cells viability upon treatment with CSA-131 (grey stripped bars) and AuP@CSA-131 (light green bars) at doses ranging from 2 to 50 µg/mL for 24, 48 and 72 h. Short dashed lines and solid lines indicate dose–response fitted curves for CSA-131 and AuP@CSA-131, respectively (**A**). An average survival of SKOV-3 cells upon CSA-131 (grey dots) and AuP@CSA-131 (blue dots) exposure (**B**). Inhibitory concentrations (IC) at 50%, 75% and 90% effects levels recorded for CSA-131 (grey stripped bars) and AuP@CSA-131 (light green bars) after 24, 48 and 72 h (**C**). Morphological alterations and decreases in cellular confluency of SKOV-3 cells treated with CSA-131 and AuP@CSA-131 at dose of 5 µg/mL for 72 h (**D**). Scale bar ~300 µm. For (**A**–**C**), results are presented as mean ± SD from three independent measurements. For (**D**), one representative result is shown. * and ^ indicate statistical significance (*p* < 0.05) when compared with control (0 µg/mL) and CSA-131-treated cultures, respectively.

In the present study, the in vitro cytotoxic effects of ceragenin CSA-131 and AuP@CSA-131 were evaluated against SKOV-3 cells at a concentration ranging from 2 to 50 µg/mL. As demonstrated in Figure 2A, exposure of ovarian cancer cells to ceragenin CSA-131 in both free-form and as conjugated with gold nanopeanuts resulted in a dose- and time-dependent decrease in SKOV-3 cells survival, with total eradication of cancerous cells at dose of 50 µg/mL. Comparative analysis confirmed the rationality of using gold nanopeanuts as a carrier for ceragenin CSA-131. Notably, at each concentration and time interval tested, AuP@CSA-131 exerted stronger inhibitory activity than unbound ceragenin, which was reflected by the significantly lower absorbance values that were recorded (Figure 2A).

To better quantitively analyze the interactions between tested compounds, the relative inhibitory effect (RIE) was calculated based on recorded survival rates. As shown in Table 1, attachment of CSA-131 to the surface of gold nanopeanuts resulted in an additional decrease in cancer survival up 43.88 ± 5.64%, which strongly supports the usefulness of AuP NPs-based nanocarrier in CSA-131 delivery. Accordingly, the average survival of SKOV-3 cells at each time-point was lower for AuP@CSA-131-treated samples than those exposed to unmodified ceragenin (Figure 2B). Employment of gold nanopeanuts as a drug carrier also resulted in a decrease in the inhibitory concentrations (IC) of the tested compounds. More precisely, inhibitory doses of AuP@CSA-131 at 50%, 75% and 90% effect levels were nearly 2-fold (1.51–2.45) lower than those noted for CSA-131 (Figure 2C). This observation was also further corroborated by phase-contrast microscopic examination of SKOV-3 cellular morphology upon exposure to tested compounds. As expected, the number of treated cancer cells was considerably lower when compared with untreated control, and cells exhibited a number of morphological alterations, including less adhesion to the culture flask, less cell spreading and observable vacuolization in cytoplasm—an effect that was far more prominent in AuP@CSA-131-treated cells (Figure 2D). Notably, the nanosystem based on spherical nanogold did not reach such effectiveness in a comparable concentration range. Collectively, it is reasonable to conclude that the attachment of ceragenin CSA-131 to the surface of gold nanopeanuts resulted in an improved cytotoxic effect against ovarian carcinoma at relatively lower concentrations. Based on this preliminary result, and having in mind our previously obtained data demonstrating the considerable toxicity of CSA-131 at concentrations higher than 20 µg/mL [33], we decided to choose the doses of 2, 5 and 10 µg/mL of the tested compounds for further experimentation. An incubation time of 72 h was also selected as being an appropriate treatment time in subsequent experiments.

**Table 1:** Relative inhibitory effect (RIE) for AuP@CSA-131 recorded at concentration range 2–50 µg/mL against human ovarian adenocarcinoma SKOV-3 cells.

(µg/mL)	CSA-131			AuP@CSA-131		
	Cell Growth Inhibition (%)			Relative Inhibitory Effect (%)		
	24 h	48 h	72 h	24 h	48 h	72 h
2	−2.01 ± 6.85	14.40 ± 1.02	16.25 ± 4.41	+25.60 ± 22.12	+7.39 ± 3.61	+1.28 ± 11.20
5	21.80 ± 9.09	16.66 ± 3.50	30.89 ± 1.89	+8.51 ± 8.68	+3.76 ± 1.09	+21.84 ± 5.45
10	34.13 ± 13.34	32.57 ± 9.04	41.51 ± 9.66	+23.81 ± 14.79	+14.05 ± 12.69	+43.88 ± 5.64
20	45.22 ± 5.75	49.71 ± 3.24	61.01 ± 2.06	+27.68 ± 9.16	+27.54 ± 9.04	+35.41 ± 3.71
50	80.81 ± 2.40	91.18 ± 4.01	100 ± 0	+15.61 ± 2.89	+8.81 ± 4.01	0 ± 0

### 3.3. AuP@CSA-131 Induces Both Apoptosis and Autophagy in SKOV-3 Cancer Cells to a Greater Extent than Unbound Ceragenin

**Figure 3:**
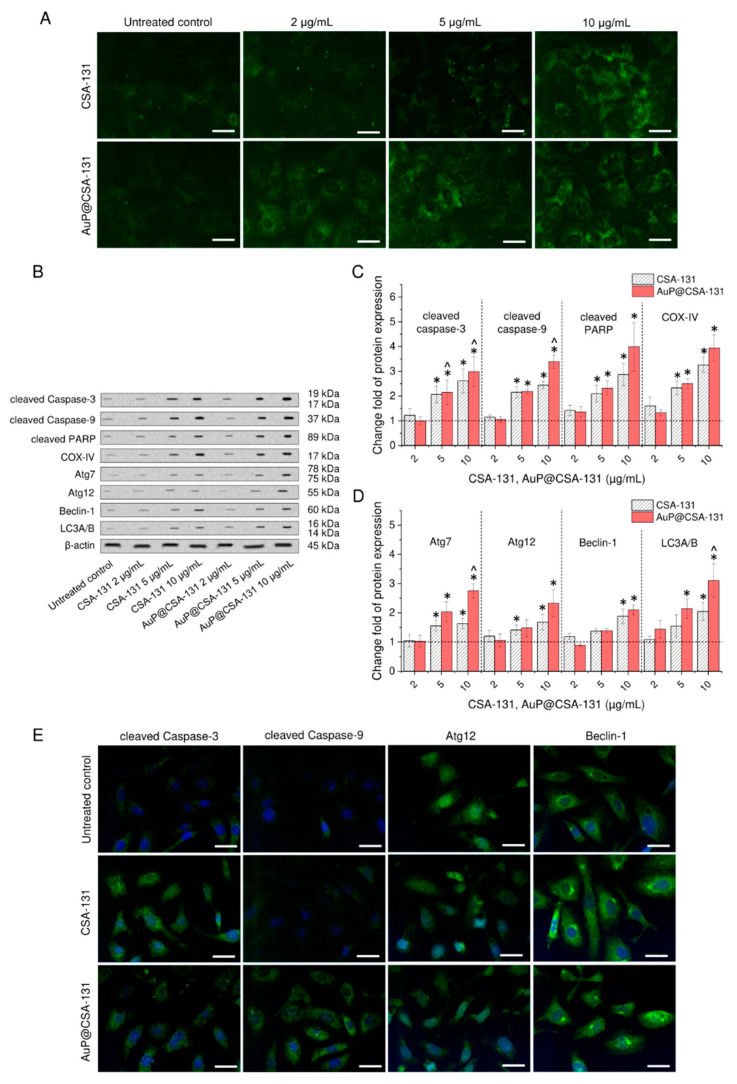
Induction of caspase-dependent apoptosis and autophagy in ovarian cancer SKOV-3 cells treated with ceragenin CSA-131 and AuP@CSA-131. Immunofluorescence staining of annexin A1 in SKOV-3 cancer cells exposed to CSA-131 and AuP@CSA-131 at doses of 2, 5 and 10 µg/mL for 72 h (**A**). Scale bar ~10 µm. Western blot measurement of alterations in apoptotic (cleaved caspase-3, cleaved caspase-9, cleaved PARP and COX IV) and autophagic proteins (Atg7, Atg12, Beclin-1 and LC3A/B) upon treatment with CSA-131 and AuP@CSA-131 at doses of 2, 5 and 10 µg/mL for 72 h. Representative results of Western blot analysis are presented in (**B**), while densitometry analysis of detected alteration is presented in (**C**,**D**). Immunostaining of apoptosis- (cleaved caspase-3 and cleaved caspase-9) and autophagy-associated proteins (Atg12 and Beclin-1) in SKOV-3 cancer cells upon exposure to CSA-131 and AuP@CSA-131 at dose of 5 µg/mL for 72 h (**E**). Scale bar ~10 µm. For (**A**,**B**,**E**), one representative result is shown. For (**C**,**D**), mean ± SD from three individual experiments is presented. * and ^ indicate statistical significance (*p* < 0.05) when compared with control (0 µg/mL) and CSA-131-treated cultures, respectively.

To investigate the killing mechanisms of the tested compounds and to check whether immobilization of CSA-131 on the surface of gold nanopeanuts might contribute to further intensification of cell death processes at comparable doses of ceragenin, we performed an analysis of the expression of selected apoptosis-related proteins using Western blot and immunofluorescence staining. Initially, expression of annexin A1 (ANXA1), being a phospholipid-binding protein colocalizing with phosphatidylserine on the outer plasma membrane leaflet [40], was elucidated. As demonstrated in Figure 3A, with increasing concentrations of CSA-131 and AuP@CSA-131, the ANXA1-derived signal rises, which suggests that SKOV-3 cells treated with the tested compounds are introduced to an apoptotic cell-killing mechanism. Notably, this effect was far more prominent in samples treated with AuP NPs-modified ceragenin. Although at doses of 2 and 5 µg/mL of CSA-131, no considerable response was detected, in samples treated with AuP@CSA-131, expression of ANXA1 increased considerably. 

To more quantitively examine this effect, alterations in apoptosis-related proteins were investigated using Western blot (Figure 3B and Figure S1). Densitometry analysis revealed that exposure to CSA-131 and AuP@CSA-131 increases the cleavage of effectors caspases—caspase-3 and caspase-9—2.52- and 3.18-fold, respectively. Simultaneously, cleavage of PARP, as well as expression of COX-IV in treated cancer cells increased by 186% and 226%, respectively, upon treatment with ceragenin CSA-131. Further increase in PARP and COX-IV by an extra 118% and 68% was possible using treatment with 10 µg/mL of AuP@CSA-131 (Figure 3C). To test whether ceragenin CSA-131 and its nanogold counterpart were involved in stimulation of autophagy in ovarian cancer cells, analysis of well-recognized autophagic proteins, including Atg7, Atg12, Beclin-1 and LC3A/B complex was performed. Similar to measurements of apoptotic-related proteins, SKOV-3 cells did not respond considerably upon treatment with CSA-131 and AuP@CSA-131 at a dose of 2 µg/mL, but treatment required higher concentrations of the tested agents to notably alter the expression of signaling factors. As demonstrated in Figure 3D, expression of Atg7, Atg12, Beclin-1 and LC3A/B when compared with control cells rose a times fold of 1.63 ± 0.18, 1.68 ± 0.26, 1.88 ± 0.25 and 2.05 ± 0.31, respectively, upon treatment with CSA-131. An increase by a further 112%, 65%, 22% and 106% was recorded when nanogold-ceragenin conjugate was tested.

In agreement with the above observations, a higher signal from immunostained apoptotic and autophagy proteins was recorded using a confocal scanning fluorescence microscope (Figure 3E). In contrast with control cells characterized by very weak or no detectable signal from tested apoptosis and autophagy markers, SKOV-3 cells treated with CSA-131 and AuP@CSA-131 exhibited strong response upon treatment with 5 µg/mL of the tested agents. Importantly, in cells exposed to the developed nanoformulation, this effect was more pronounced, which is in agreement with the cytotoxicity tests. These results indicate that both CSA-131 and AuP@CSA-131 initiated apoptosis and autophagy in cancer cells and that gold nanopeanunts-CSA-31 conjugate exerted a higher death induction efficiency in comparison with free ceragenin.

### 3.4. Anti-Oxidant Protective Mechanisms of Cells Are Disrupted upon Exposure to AuP@CSA-131

**Figure 4:**
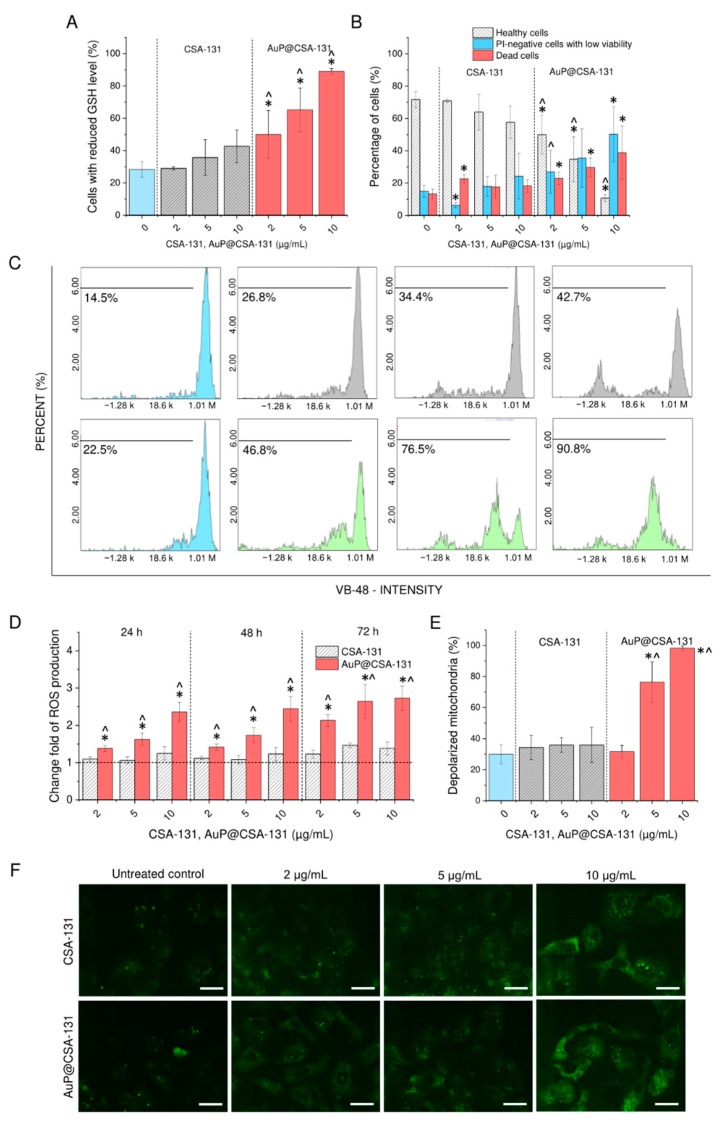
Disruption of anti-ROS protective mechanisms in SKOV-3 cancer cells treated with CSA-131 and AuP@CSA-131. The number of cells with reduced GSH levels recorded after 72 h incubation with CSA-131 (grey stripped bars) and AuP@CSA-131 (light green bars) at doses of 2, 5 and 10 µg/mL when compared with control samples (light blue bar) (**A**). Percentage representation of healthy cells (grey bars), PI-negative cells with low viability (light blue bars) and dead cells (pink bars) upon exposure to CSA-131 and AuP@CSA-131 at doses of 2, 5 and 10 µg/mL for 72 h (**B**). Representative flow cytometry plots for GSH intracellular measurements (**C**). Induction of reactive oxygen species production upon treatment with CSA-131 (stripped grey bars) and AuP@CSA-131 (red bars) at doses of 2, 5 and 10 µg/mL for 24, 48 and 72 h (**D**). The percentage of SKOV-3 with depolarized mitochondria as an effect of CSA-131 (stripped grey bars) and AuP@CSA-131 (red bars) treatment when compared with untreated control (light blue bar) (**E**). Immunofluorescence staining of NADPH subunit 4 in SKOV-3 cancer cells exposed to CSA-131 and AuP@CSA-131 at doses of 2, 5 and 10 µg/mL for 72 h (**F**). Scale bar ~10 µm. For (**A**,**B**,**D**,**E**), results are presented as mean ± SD from three experiments. For (**C**,**F**), one representative result is shown. * and ^ indicate statistical significance (*p* < 0.05) when compared with control (0 µg/mL) and CSA-131-treated cultures, respectively.

In the next step of the research, we examined the possibility of inducing oxidative stress in ovarian cancer cells upon treatment with CSA-131 and AuP@CSA-131 at doses up to 10 µg/mL. A high level of intracellular free thiols, including reduced glutathione (GSH), is recognized as a most critical protective mechanism, shielding cells from oxidative damage and toxicity from ROS inducers [41]. In effect, a declining intracellular concentration of GSH is a favorable approach for killing cancer cells [42]. In this study, we demonstrated that ceragenin CSA-131 is able to decrease GSH levels at a dose of 10 µg/mL, but only when it is conjugated with a gold nanocarrier. As demonstrated in Figure 4A,C, with an increasing concentration of the tested cytotoxins, the amount of cells with reduced GSH increases. Nevertheless, when CSA-131 at a dose of 10 µg/mL was applied, only slight a response was observed (42.67 ± 10.21% vs. 28.33 ± 4.93% in control cells), and this result did not reach statistical significance. In contrast with this, treatment with AuP@CSA-131 even at a low dose of 2 µg/mL increased the percentage of GSH-depleted cells to 50.0 ± 14.73% and to 89.7 ± 1.73% when a 10 µg/mL dose was applied. In addition to these analyses, PI/AO double staining of treated SKOV-3 cells allowed a determination of whether these GSH-affected cells were indeed dead. As demonstrated in Figure 4B, the population of treatment-affected cells was divided nearly equally between dead cells and PI-negative cells with low viability. Being in accordance with the previous tests, the number of live cells was statistically lower when AuP@CSA-131 was applied (10.75 ± 2.06% of viable cells upon treatment with AuP@CSA-131 at dose of 10 µg/mL compared with 57.66 ± 10.01% of survival in samples exposed to CSA-131 at 10 µg/mL). The above results strongly support the hypothesis that the killing ability of AuP@CSA-131 is determined by the disbalance of the redox state of cancer cells.

As expected, further analyses indicated that in treated SKOV-3 cells overproduction of reactive oxygen species occurred. Notably, this effect was highly correlated with intracellular GSH decline. For this reason, free CSA-131 at tested doses was not able to induce excessive overproduction of ROS—at the highest tested concentration, i.e., 10 µg/mL, the amount of reactive oxygen species increased only by 38%, which seemed to be insufficient to induce unbalanced oxidative stress. In contrast to that, incubation with AuP@CSA-131 at doses of 5 and 10 µg/mL resulted in a 2.7-fold increase in production of free radicals (Figure 4D). Furthermore, as demonstrated in Figure 4E,F, with increasing concentrations of the tested compounds, a continuous decrease in red JC-1-derived fluorescence signal was observed, indicating a loss of electrochemical gradient in the mitochondria of SKOV-3 cells, particularly in AuP@CSA-131-treated samples. More quantitively, the response to CSA-131-mediated treatment was not considerable—the number of cells affected by treatment rose by only 6% when compared with the control populations. In contrast, when cells were exposed to AuP@CSA-131 at doses of 5 and 10 µg/mL, this index increased to 76.33 ± 13.01% and 98.33 ± 1.53%, respectively, which indicates a far better response to treatment with nanogold conjugate than free CSA-131 (Figure 4E). These results were also confirmed by the highly apparent increase in NOX4 expression (Figure 4F). Collectively, the above results strongly indicate that AuP@CSA-131 induced a far stronger disturbance in the oxidative balance of SKOV-3 than free CSA-131 at comparable doses. The performed analyses also point out that the ROS-modulating ability of AuP@CSA-131 was crucial for exerting potent anti-cancer activity.

### 3.5. Formation of CSA-131-Containing Nanosystem Alters the Biodistribution of Ceragenin in Treated Animals

**Figure 5:**
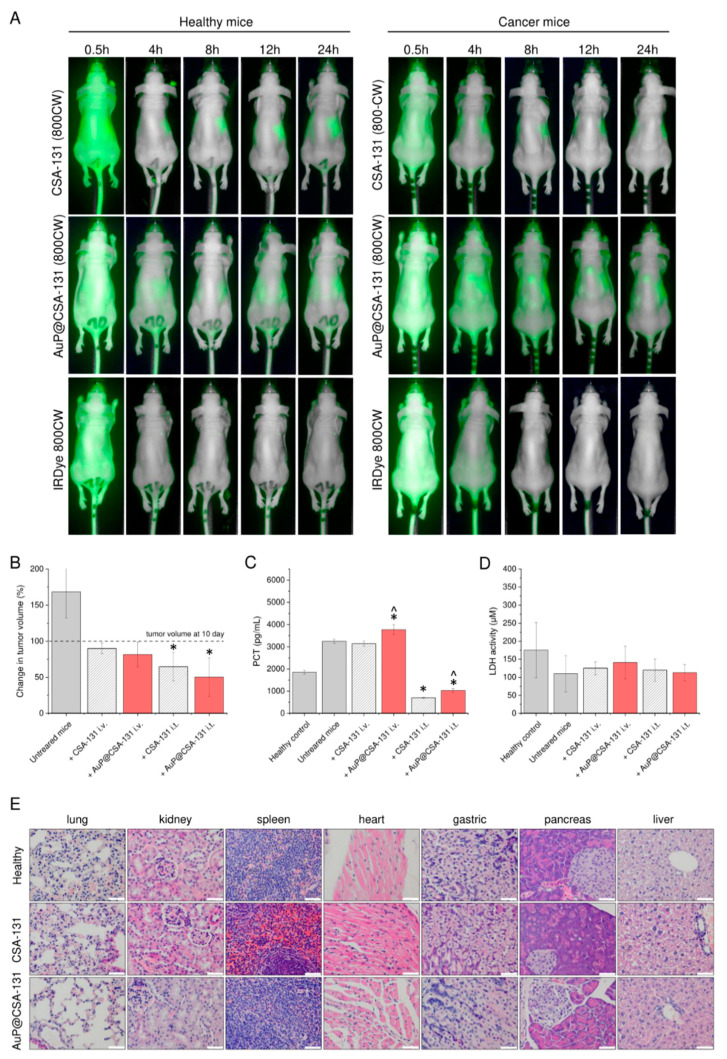
Biodistribution of exogenously administered CSA-131 labelled with IRDye^®^ 800CW (CSA-131 (800CW)), AuP@CSA-131 labelled with IRDye^®^ 800CW (AuP@CSA131 (800CW)) and unmodified IRDye^®^ 800CW dye estimated by fluorescence-based analysis of fluorescence signal in healthy and cancer-bearing animals (**A**). Changes in volume of tumors collected from untreated animals and mice treated with CSA-131 (stripped grey bars) or AuP@CSA-131 (red bars) administered intravenously or intratumorally when compared with day 10 (beginning of treatment) (**B**). Concentrations of procalcitonin (PCT) and lactate dehydrogenase (LDH) recorded in serum of sacrificed animals (**C**,**D**), respectively. Histological analysis of the main organs of healthy animals as well as CSA-131 and AuP@CSA-131-treated animals carried out using hematoxylin–eosin staining (200×). White scale bar indicates 250 µm. Results from one representative experiment are shown. For (**A**,**E**), one representative result is shown. For (**B**–**D**), results are presented as mean ± SD from three experiments. * and ^ indicate statistical significance (*p* < 0.05) when compared with control, cancer-suffering animals (0 µg/mL) and CSA-131-treated mice, respectively.

The anti-tumor efficiency of the developed nanosystem was determined in a pre-clinical mouse xenograft model of ovarian cancer, induced by subcutaneous injection of SKOV-3 cells into immune-deficient mice. Before starting the testing, animals were divided into experimental groups, as indicated in Table 2. Biodistribution of intravenously administered CSA-131 and AuP@CSA-131 was explored by their labelling with IRDye^®^ 800CW, a fluorescent dye, which was detected by the 800 nm channel using the Pearl^®^ Trilogy small animal imaging system. As shown in Figure 5A, upon i.v. administration into healthy mice, CSA-131 and AuP@CSA-131 was concentrated mainly in the liver and kidneys. Although both tested compounds seemed to have a similar biodistribution profile and were mainly excreted by the kidneys and the liver, a considerable alteration in the blood clearance among animal groups was observed. More precisely, AuP@CSA-131 was noted to be removed from the bloodstream at a slower rate than unmodified ceragenin, i.e., it located in the liver and kidneys after approximately 8 h, while CSA-131 was located after 4 h. Based on the above results, it is justified to state that immobilization of ceragenin on the surface nanoparticles might help to control the pharmacokinetics of CSAs.

### 3.6. Administration of CSA-131 and AuP@CSA-131 Suppresses the Growth of Ovarian Tumor and Decreases Procalcitonin Concentration with Limited Toxicity to Treated Animals

To quantitatively analyze the anti-tumor activity of the tested formulation, both CSA-131 and AuP@CSA-131 were administered intravenously or intratumorally every 72 h starting from day 10, when tumors had grown to an appropriate volume. On the last day of the experiment, the volume of grown tumors was compared across formulations. As demonstrated in Figure 5B, the volume of the ovarian tumors increased over time in the control animals, whereas it was suppressed by treatment with CSA-131 and AuP@CSA-131, with a higher suppression observed for AuP NPs-based nanoformulation and when the drug was applied intratumorally. More specifically, in the control group of sterile saline-treated animals, the volume of tumors increased by 68.31 ± 36.11% between day 10 and day 29, while in treated animals, the growth was completely inhibited and tumors decreased their volume further from 10.1% (for CSA-131 i.v.) to 49.7% (for AuP@CSA-131 i.t.) when compared with day 10. Notably, intratumoral administration of the tested agents was recorded to be more efficient for tumor growth suppression. To indirectly confirm the recorded observation, we measured the concentration of procalcitonin, it being a biomarker of cancer development [43,44], in the serum of sacrificed animals. According to data presented in Figure 5C, tumor growth in untreated animals corelated with elevated concentrations of PCT (3250 ± 94.25 pg/mL) when compared with healthy animals (1855 ± 81 pg/mL). No significant effect of intravenously administered agents was recorded. In contrast with that, injection of CSA-131 and AuP@CSA-131 into the tumor resulted in a 4.62- and 3.16-fold decrease in PCT serum concentration, highlighting the anti-cancer efficiency of those compounds upon intratumoral administration. Importantly, treatment of cancer-bearing mice did not cause any significant systemic toxicity. We did not observe any statistically significant differences between tested animal groups when serum LDH concentration was measured (Figure 5D). As an additional confirmation of the lack of toxicity, we assessed by histopathological analysis the changes in the organs of mice intravenously injected with CSA-131 and AuP @ CSA-131. We did not observe any pathological differences in lung, kidney, spleen, heart, gastric system, pancreas and liver tissues among the groups (Figure 5E). No deaths were observed in any of the treatment groups. During the study, animals from all study groups were in good health and behavioral condition, no pain symptoms or significant weight loss were observed. The animals were active, consuming food and water properly. The only disturbing symptom observed by the team conducting the experiment was scabs on the surface of the tumor in animals receiving the drug intratumorally. No pathological changes in the morphology of the organs were found during the necropsy.

### 3.7. Both Ceragenin CSA-131 and AuP@CSA-131 Nanosystem Modulate the Immune Response in Cancer-Bearing Mice

**Figure 6:**
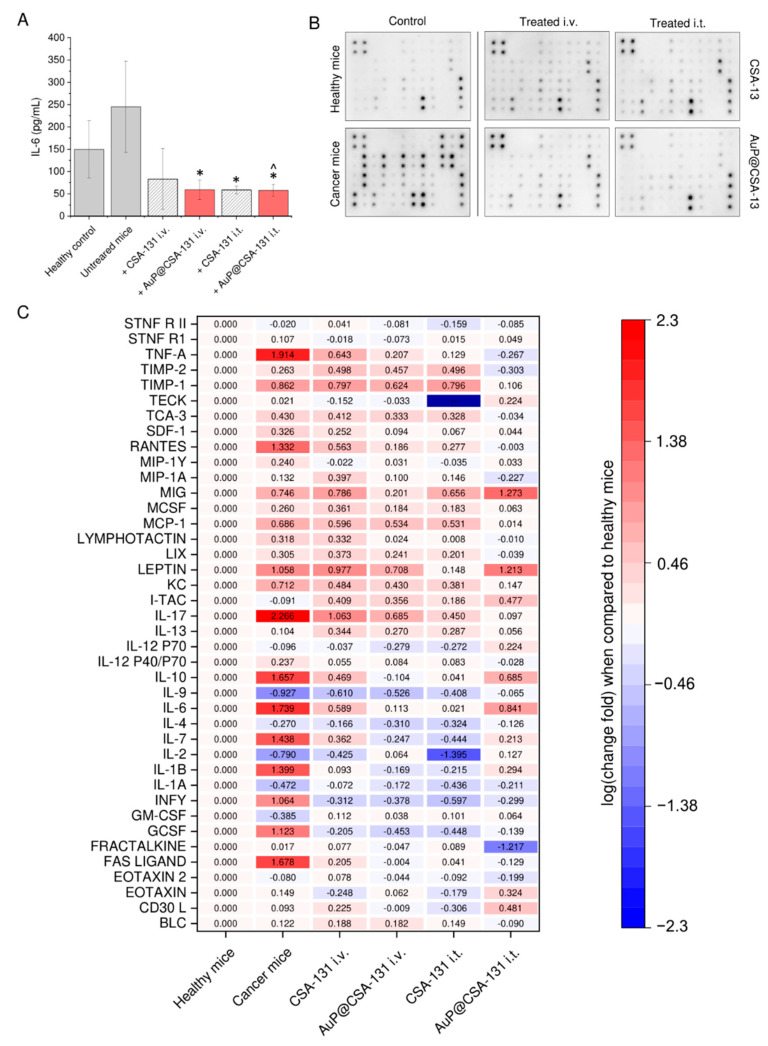
Blood plasma levels of IL-6 recorded in serum samples collected from control and both healthy and cancer-bearing animals (grey bars), as well as those treated with CSA-131 (stripped grey bars) or AuP@CSA-131 (red bars), both intravenously and intratumorally (**A**). Membrane microarray-based analysis of changes in serum content of inflammation-associated cytokines and growth factors. Representative image of membranes collected upon chemiluminescence analysis (**B**). Quantitative compilation of changes in concentration of cytokines and growth factors in analyzed animal groups. Red and blue colors indicate fold increase and decrease in serum levels of tested analytes when compared with healthy, control animals (**C**). For (**A**,**C**), results are presented as mean ± SD from three experiments or two duplicates, respectively. * and ^ indicate statistical significance (*p* < 0.05) when compared with control, cancer-suffering animals (0 µg/mL) and CSA-131-treated mice, respectively.

Some previous studies suggest that ceragenins, apart from direct cytotoxic mechanisms, might display a spectrum of indirect effects, such as modulation of immune responses [45]. Our data, obtained from analysis of serum collected from ovarian cancer-bearing mice treated with CSA-131 and AuP@CSA-131 reveal that administration of compounds, both intravenously and intratumorally, altered the serum IL-6 profile by decreasing its concentration when compared with tumor-bearing animals (Figure 6A). Based on this preliminary result, we performed an expanded microarray-based analysis that semi-quantitatively profiled the changes in serum levels of inflammatory cytokines. According to the results presented in Figure 6B,C, in serum collected from animals with an induced ovarian tumor, the profile of pro-inflammatory factors was considerably altered, presenting an aggressive phenotype, while in serum collected from treated animals, the concentrations of tested cytokines were similar to those in the control group or even lower. More precisely, tumor-bearing animals were characterized by a considerable increase in proinflammatory cytokines, chemokines and growth factors, which are considered to be factors that predict worse outcome in cancer patients. Particularly, TNF-α, RANTES, IL-1β, IL-6, IL-7, IL-10, IL-17, GCSF, Fas ligand and IFN-γ were considerably altered when compared with control mice (>1 log of change). At the same time, factors that are well-recognized as immunosuppressive and cancer growth-inhibiting (i.e., IL-2 and IL-9) were downregulated (<−0.5 log of change). As a result of the performed treatment, it was recorded that concentrations of the indicated factors returned to levels noted for healthy animals. This beneficial tendency was prominent, particularly for intratumorally administered agents.

## 4. Discussion

The attachment of anti-cancer agents to nanoparticle-based drug delivery carriers is a favorable approach for decreasing effective doses of antineoplastic compounds, thus limiting their systemic toxicity and achieving enhanced therapeutic efficiency at the same time [4]. In present work we aimed to improve the anti-cancer effectiveness of ceragenin CSA-131 by its immobilization on the surface of gold nanopeanuts, which allowed us to exert potent activity at doses of CSA-131, which are recognized as being non-toxic to non-cancerous cells. Notably, the membrane-permeabilizing properties of ceragenins, which directly determine the antimicrobial and anti-cancer effects of these compounds, are also a crucial reason for their toxicity [31], and the relatively low hemocompatibility of ceragenins is presently recognized as a critical limitation hampering their systemic administration. As we assessed previously, ceragenins exert considerable hemolytic activity in a concentration range of 20–100 µg/mL [31]. In the case of bacterial and fungal infections, significantly lower ceragenin concentrations, usually not exceeding 5–10 µg/mL, are required [32,46,47], which ensures the safety of their use in such a clinical application. At the same time, the effective inhibition of cancer cells’ viability is achievable at higher ceragenin doses, which are often hemolytic and toxic to host cells (Figure 2A). For this purpose, the optimization of ceragenins with regard to their efficiency/safety ratio is required. As we demonstrated, effective inhibitory concentrations of AuP@CSA-131 are 2 times lower than those reported for CSA-131 (Figure 2C), which strongly supports the hypothesis that an appropriate effectiveness of ceragenins might be obtained by their incorporation with metallic nanocarriers.

So far, modulation of the membrane-permeabilizing properties of ceragenins due to their incorporation on the surface of nanomaterials was explored by us using mostly spherical-shaped iron oxide nanoparticles as drug nanocarriers [23,27,30,48]. Notably, however, an ever-growing body of evidence has demonstrated that gold nanoparticles, particularly those with non-spherical morphology, exert a number of advantages over magnetic, iron oxide-based nanostructures [15]. Particularly, the employment of nanoparticles in a shape other than a sphere offers the possibility of loading more molecules on the particle surface, which is thus converted into an enhanced concentration of anti-cancer agents targeting the desired cancerous tissues [15]. Another advantage of an elongated-type of gold NPs is also their reduced clearance and prolonged blood circulation times in comparison with their spherical counterparts [16]. For this reason, our latest efforts have focused on the synthesis of effective ceragenin-based nanosystems consisting of non-spherical gold nanoparticle as a metallic core. So far, the validity of such an approach has been confirmed in microbiological in vitro models [49]. In this study, we aimed to elucidate how the employment of gold nanopeanuts as drug nanocarriers might modulate the anti-cancer effectiveness of CSA-131 and to explore the mechanisms of their activity. In general, elongated nanoparticles are recognized as being more cytotoxic than nanospheres of comparable size and dose [17,18]. In the majority of reported studies, gold nanorods were also characterized by higher cellular uptake efficiency, thus presenting an improved drug delivery efficiency when compared with spherical ones [50,51]. Indeed, our previous research revealed that peanut-shaped gold nanoparticles are far more effective against cancer cells than those spherical ones ([22]; see Supplementary Materials). Moreover, a nanosystem consisting of CSA-131 attached to the surface of spherical-shaped gold NPs was not efficient enough against ovarian cancer cells.

When analyzing the mechanism of cytotoxicity of CSA-131 and AuP@CSA-131, it was noted that both of these compounds induce caspase-dependent apoptosis in ovarian cancer cells, with a more pronounced effect for the ceragenin-nanopeanuts formulation (Figure 3A). Indeed, this cell death type was noted previously in ceragenin-treated cancers; however, available data are inconsistent when it comes to describing the pathways necessary for its initiation [28,29]. Previously, Kuroda et al. reported that ceragenin CSA-13 exerts antiproliferative effects in colon cancer HCT116 cells, which is determined by cell cycle arrest at the G1/S phase and induction of caspase-independent apoptosis [29]. Conversely, our previous study performed using breast cancer MCF-7 cells revealed a critical involvement of caspases and mitochondrial functioning disturbance in apoptotic cell death [30]. In line with this, we demonstrated that both the ceragenin CSA-131 and ceragenin-containing nanosystem induced caspase-dependent apoptosis, as evidenced by the cleavage of effectors caspases—caspase-3 and caspase-9 (Figure 3B,C). What is more interesting is that a concurrent activation of the autophagy process occurs in treated SKOV-3 cells, as was characterized by the enhanced expression of Atg7, Atg12, Beclin-1 and LC3A/B proteins (Figure 4). Autophagy, despite its pro-survival role in a variety of tissues, is often co-induced by the apoptosis signaling pathway upon exposure to anti-cancer agents [52,53]. Although autophagy may serve the development of chemoresistance [54], overstimulation of this pathway leads to an extensive cellular response and the introduction of a cell to a cell death-type II mechanism, resulting in degradation of cellular compounds and organelles [55]. Particularly, such response occurs often upon treatment of cancers with ROS modulators, including gold nanoparticles [56] and membrane-active compounds [57]. Although the ceragenin-mediated initiation of the apoptosis process is relatively known [28,29,30], the activation of autophagy by CSAs has not been reported, according to our best knowledge. To date, only a few observations have been made in relation to LL-37, an antimicrobial peptide from the cathelicidin family, which is recognized as an original model whose amphipathic chemical character and membrane mechanism of action is mimicked by ceragenins [58]. In such a context, LL-37 peptide was reported to upregulate the autophagy-related gene expression in macrophages and promote formations of autophagosomes to enhance the killing of intracellular bacteria [59,60]. In anti-cancer therapies, FK-16 peptide (corresponding to residues 17–32 of LL-37 peptide) induced autophagic cell death in colon carcinoma through the p53-Bcl-2/Bax cascade [57]. The above observations might suggest that ceragenins, apart from mimicking the membrane-permeabilizing properties of antimicrobial peptides, may also activate similar signaling pathways, very likely by activation of some cell membrane receptor. Importantly, compelling evidence indicates that over-stimulation of autophagy-related proteins over a certain physiological level changes the direction of the cellular role of autophagy from protective and pro-survival to pro-death [61]. For instance, such autophagic cellular degradation in non-small-cell lung cancer cells upon gold nanoparticles exposure was presented by Ke et al. [62]. Autophagy was also noted to be implicated in cellular killing by peanut-shaped gold nanoparticles, as we demonstrated most recently [22]. Based on this, we suggest that the improved killing activity of AuP@CSA-131 is determined by the enhanced uptake of ceragenin into cancer cells, which leads to autophagy over-stimulation, thus subsequently promoting steps that lead to the death of ovarian cancer cells. There is also a possibility that increased induction of apoptosis and autophagy processes is determined by synergistic anti-cancer activities of gold nanopeanuts and ceragenin; however, we do not believe that it occurred in our experimental setting. Notably, the amount of gold nanoparticles present in the CSA-131-containing nanosystem (less than 15 pg/mL, corresponding to 10 µg/mL of AuP@CSA-131 as calculated based on the ceragenin loading rate) was too little to exert any cancer inhibitory effect, according to our previously published data using the same cell line [22]. In microbiological models, improved bactericidal activity of Au@CSA-131 was reported to be conditioned by an increase in the local concentration of CSA-131 on the surface of gold NPs, followed by AuP NPs-supported uptake of CSAs rather than by the antibacterial activity of the gold nanoparticles themselves [49]. As a preliminary evaluation, such a concentration of AuP NPs is also unable to activate any apoptosis-associated signaling pathways or to alter cytokines release from cancer cells, which was also the reason why we did not test AuP NPs in the animal model.

When exploring the mechanism of recorded cytotoxicity, disruption of anti-ROS protective mechanisms (Figure 4A–C) and the occurrence of excessive oxidative stress with subsequent mitochondrial functioning interruption (Figure 4D–F) were noted to be highly involved in the AuP@CSA-131-mediated anti-cancer effect. In our previous studies, we observed that ceragenins considerably affected the physiological balance between reduced and oxidized thiols, leading to a decline in GSH intracellular levels and an abolition of the anti-ROS protective mechanisms of cancer cells. In effect, a loss of mitochondrial potential and overproduction of free radicals occur, which ultimately direct the cell into death pathways [30,33]. In agreement with those reports, we demonstrate here that a similar mechanism of action took place in SKOV-3 cells treated with ceragenin-conjugated gold nanopeanuts (Figure 4). We presume that GSH reduction was CSA-131-mediated exclusively and that the increased effect recorded for AuP@CSA-131 resulted from improved delivery of this compound to the cancer cells and an increase in its intracellular concentration. Admittedly, there are a compelling number of studies indicating that gold nanoparticles exert anti-cancer activity due to an interaction with GSH, which in effect shifts the cellular cytosol from a reducing to an oxidizing environment [22,63,64]. More recently, we demonstrated this mechanism for peanut-shaped gold nanoparticles, them being a ceragenin nanocarrier in our nanosystem [22]. The formation of ROS upon laser excitation is also the basis for photothermal therapy, for which gold nanoparticles seem to have the greatest clinical application [65]. Nevertheless, as we stated before, the amount of AuP NPs applied together with CSA-131 in the form of a nanosystem, was too low to achieve such effect.

Apart from an intensification of CSA-131 anti-cancer activity by carrying the compound into the interior of cancer cells, gold nanoparticles were noted to considerably alter the biodistribution of ceragenin (Figure 5A). In agreement with our previous observations regarding CSA-13 biodistribution [66], we recognized that both CSA-131 and AuP@CSA-131 are mainly excreted by the kidneys and the liver, with delayed clearance in cancer-bearing animals. We assume that this prolongation of CSA-131/AuP@CSA-131 blood half-life in tumor-bearing mice resulted from metabolism changes upon aging of the animals and cancer development, rather than because of the direct effect of the applied compounds since extended elimination was also noted for IRDye^®^ 800CW, which is well-recognized as a non-toxic probe in animal studies [67]. Importantly, we recorded considerable differences in the biological half-life and circulation residence times between CSA-131 and AuP@CSA-131. As demonstrated, the developed nanosystem residues in the mouse bloodstream lasted approximately 4 h longer than unfunctionalized ceragenin, as evidenced by the delayed loss of a compound-derived signal in the animals′ circulation (Figure 5A). An undeniable amount of evidence demonstrates that the development of antineoplastic-containing nanosystems is a favorable approach for modulating the biodistribution and for controlling the pharmacokinetic parameters of applied therapeutics. For instance, paclitaxel and ceramide incorporated with polymer-blend nanoparticles obtained higher concentrations in the blood of breast cancer-bearing animals when compared with free drugs, which was achievable due to a reduction in systemic clearance and a longer retention time [68]. In another study, improved drug retention was noted for paclitaxel-loaded polymeric micelles [69]. Decreased in vivo biodegradation, liver distribution and urinal excretion are also characteristic of unfunctionalized rod-like nanoparticles themselves [70]. Based on this, we assume that the attachment of ceragenin to the surface of AuP NPs is critical for the modulation of ceragenin pharmacokinetics. Expectedly, this extended blood circulation might be translated into improved uptake of the anti-cancer compound by the ovarian tumor, which would be reflected in a decreased volume of malignant tissue (Figure 5B). Moreover, such variation of biodistribution also allows the exertion of prolonged systemic effects, such as immunomodulation. As demonstrated, treatment of tumor-bearing animals resulted in moving the serum inflammatory profile to a less severe level, decreasing the content of pro-inflammatory cytokines, and elevating those immunosuppressive and anti-inflammatory ones (Figure 6C), all of which is favorable for inhibiting tumor growth and limiting cancer cell division. It is well-known that pro-inflammatory cytokines, particularly IL-1β, IL-6 or TNF-α, promote ovarian tumor growth and metastasis, and their high serum levels are correlated with poor clinical outcomes [71,72]. Improvement in the inflammatory profile, as demonstrated in this study would be highly beneficial for effective cancer treatment. Naturally, recorded changes partially reflect treatment progress, and observed drops in level are associated mostly with limited cancer burden. Interestingly, however, serum concentrations of some factors—particularly granulocyte colony-stimulating factor (GCSF), interferon γ (IFN-γ), IL-1β, IL-6 or IL-12 P70—in treated animals are even lower than those recorded in healthy, control animals (Figure 6). It can be assumed that both CSA-131 and AuP@CSA-131 are able to control the inflammatory profile of animals, despite the lack of systemic disease. For instance, it is well-recognized that aged mice are characterized by age-related alteration in the inflammatory response [73,74] and that the animals that were tested at the end of the experiment were 16–18 weeks old. This might indicate that CSA-131 and AuP@CSA-131 exert anti-cancer effects by additional indirect mechanisms related to the modulation of factors that affect inflammatory response, tumor growth and cancer dissemination. Although these results are preliminary and require confirmation in more quantitative analyses, they strongly suggest that ceragenins (and more actively, CSAs-based nanosystems) might be considered as anti-cancer agents, acting both locally and systemically. Nevertheless, there is a limited amount of data on the immunomodulatory properties of ceragenins and CSA-containing nanosystems, while no research has been performed to elucidate the impact of CSAs on the expression of angiogenic factors. For this reason, the presented data should be more thoroughly investigated in modified experimental settings. A question is also left open as to whether such CSA-AuP NPs conjugate after appropriate modification that might be effectively used as an improved photothermal therapy compound since gold nanorods, reflecting the majority of morphological features of gold nanopeanuts, are reported to have an improved absorption band at NIR light when compared with other Au NPs, and as such are used as photothermal nanodevices for the release of biomolecules, e.g., doxorubicin [75]. However, an examination of this hypothesis requires modified and more comprehensive in vivo model analyses.

Although we believe that our results are promising for the further development of ceragenin-containing nanoformulations, some limitations of this study should also be recognized. First, our research was performed using only one ovarian cancer cell line, and while ceragenins and CSA-based nanosystems were previously described as being effective in vitro against different cancer cell lines, including those cell lines derived from breast, lung and colon [28,29,30,33], it should be pointed out that those results were not confirmed in animal models. For this reason, there is a possibility that the genetic diversity of ovarian tumors might considerably affect the anti-cancer activity of ceragenins and CSA-containing nanosystems. A question is also left open regarding whether the effectiveness of the tested compound would vary if the observations were to be carried out for longer than 28 days, a time at which drug resistance could potentially be induced. The reduction in the sensitivity of cancer cells to prolonged administration of ceragenins and CSA-based nanoformulations has not been analyzed so far in either cellular or animal models, and it is certainly a topic worthy of further research. Second, we did not include any non-malignant cell lines in our study (such as ovarian epithelium cells), which might raise a question about the safety of the applied treatment. Notably, the achievement of satisfactory therapeutic efficiency in patients undergoing anti-cancer treatment, while simultaneously sparing healthy cells, is a challenge for modern chemotherapy. Although a compelling amount of data demonstrates that cancer cells are characterized by a spectrum of cell-specific features, such as elevated negative charges on their surface, enhanced fluidity and increased surface area, all of which make them more susceptible to treatment with membrane-targeting agents [27], we cannot rule out the possibility that treatment with ceragenins and CSA-based nanoagents could be harmful to non-malignant cells. This topic seems to be of great interest, given the above-described data demonstrating the prolonged circulation of AuP@CSA-131 after i.v. administration. While such modulation of biodistribution and pharmacokinetic parameters is desirable for anti-cancer therapy because it allows a greater accumulation of cytostatic effects in the tumor, the prolonged presence of the drug in the bloodstream also increases the exposure of healthy organs to this factor, which directly determines its toxicity. So far, we confirmed that intravenous administration of CSA-131 and AuP@CSA-131 does not induce toxic effects in the main organs of mice (as evidenced using histopathological staining); nevertheless, extended and more detailed analyses using serum parameters profiling might be desirable for an accurate safety assessment of such an approach. Considering that reports on the safety of administration of AuNPs into living organisms and their possible accumulation in the vital organs are still equivocal [76], some caution would be justified. Potentially, a reduction in these problems could be achieved through (i) the use of a ceragenin-based nanoformulation containing additional cancer cell-targeting molecules or (ii) applying the agent via an alternative route of administration. The first solution is supported by an ever-growing amount of evidence demonstrating that the distinctive cell surface characteristics of cancer cells compared with normal cells might be successfully exploited by therapeutic targeting. Consequently, a wide spectrum of cellular receptors and proteins, including transferrin receptor, insulin-like growth factor 1 (IGF-1) receptor, folate receptor or integrins has been widely investigated to date as factors for increasing drug selectivity against cancer cells [77]. We did not use any targeting factors in this study, but certainly this line of research is desirable in the further synthesis of improved CSA-containing nanosystems. On the other hand, an advantage of our study is its evidence that intratumoral administration of CSA-131 and AuP@CSA-131 increases the effectiveness of the applied treatment without considerable toxicity. We are aware that the injection of a cytostatic treatment directly into a tumor is a drug administration technique of less clinical importance than intravenous administration; however, the data obtained by both our team (see above) and others [78,79] strongly suggest that this route should be considered as a way to reduce the side effects of chemotherapy. This administration route may seem particularly interesting in the case of nanoparticle-based cytostatics (AuP@CSA-131 in our case) because of reports that the intravenous application of nanoparticles may cause perturbations in the coagulation system and disruption of hemostatic balance, potentially leading to disseminated intravascular coagulation (DIC) or consumptive coagulopathy [80]. During our experiment, we did not observe any symptoms of coagulation disorders in animals; however, we believe that intratumoral drug administration should be considered in future studies in order to reduce the tumor burden in animals.

## 5. Conclusions

In summary, we present data supporting the utility of newly developed gold nanopeanuts as nanocarriers for the delivery of ceragenin CSA-131 into ovarian adenocarcinoma. Two mechanisms of cancer cell eradication and tumor growth inhibition were recognized based on the collected data: (i) direct interactions involving reactive oxygen species (ROS)-mediated induction of apoptosis and autophagy processes in cancer cells and (ii) indirect limitation of pro-tumorigenic cytokine and growth factor concentrations, leading to a restrained severity of cancer burden. Combining CSA-131 and AuP NPs into core-shell nanosystems resulted in improvement in the anti-cancer activity of CSA-131, as measured by the amount required to achieve satisfactory therapeutic efficiency. The employment of such a nanoformulation also allowed a reduction in the effective doses of ceragenin to concentrations that are considered to be non-toxic, opening a window of opportunity for its systemic administration. The employment of gold nanopeanuts as CSA-131 carriers revealed a spectrum of advantages when compared with free molecules, particularly revealing their improved anti-cancer and anti-inflammatory efficiency, and more interestingly, their attractive pharmacokinetic profile. Hypothetically, the multifaceted features of gold nanoparticles can contribute to extending the application of CSA-based nanosystems as agents in photothermal/photodynamic therapy. Overall, these results provide a rationale for further development of gold nanopeanuts-based drug delivery carriers as a promising approach to cancer therapy.

## Figures and Tables

**Table 2:** Groups of animals used in animal study.

Group	Purpose of Experiment	Number of Animals	Cancer Induction	Administered Compound	Dose of Administered Compound	Route of Drug Administration
1	Biodistribution assessment	6	−	CSA-131 (800CW)	10 µg/mL (including 1 µg/mL of IRDye^®^ 800CW)	intravenously
2		6		AuP@CSA-131 (800CW)	10 µg/mL (including 1 µg/mL of IRDye^®^ 800CW)	
3		6		IRDye^®^ 800CW	1 µg/mL	
4		6	+	CSA-131 (800CW)	10 µg/mL (including 1 µg/mL of IRDye^®^ 800CW)	
5		6		AuP@CSA-131 (800CW)	10 µg/mL (including 1 µg/mL of IRDye^®^ 800CW)	
6		6		IRDye^®^ 800CW	1 µg/mL	
7	Anti-tumor efficiency evaluation	3	−	0.9% sterile saline	50 µL	intravenously
8		3	−	0.9% sterile saline	50 µL	intratumorally
9		3	+	0.9% sterile saline	50 µL	intravenously
10		3	+	0.9% sterile saline	50 µL	intratumorally
11		6	+	CSA-131	10 µg/mL (50 µL)	intravenously
12		6	+	AuP@CSA-131	10 µg/mL (50 µL)	
13		6	+	CSA-131	10 µg/mL (50 µL)	intratumorally
14		6	+	AuP@CSA-131	10 µg/mL (50 µL)	

## Data Availability

Materials described in the manuscript, including all relevant raw data, will be freely available to any scientist wishing to use them for non-commercial purposes upon request via e-mail to the corresponding author.

## Supplementary Materials

The following are available online at https://www.mdpi.com/article/10.3390/cancers13215424/s1, Figure S1: Uncropped Western Blot Figures of Figure 3B.
