# Supplementary material for: Peanut-Shaped Gold Nanoparticles with Shells of Ceragenin CSA-131 Display the Ability to Inhibit Ovarian Cancer Growth In Vitro and in a Tumor Xenograft Model
Source: Cancers (Basel). 2021 Oct 29;13(21):5424. doi: 10.3390/cancers13215424 (PMC8582422; doi:10.3390/cancers13215424)
Supplement: Supplementary file 1 [file cancers-13-05424-s001.zip › cancers-1410018-supplementary.pdf]

cancers MDPI

Supplementary materials: Peanut-Shaped Gold Nanoparticles with Shell of Ceragenin CSA-131 Display Ability to Inhibit Ovarian Cancer Growth in Vitro and in a Tumor Xenograft Model

Ewelina Piktel, Ilona Ościłowska, Łukasz Suprewicz, Joanna Depciuch, Natalia Marcińczyk, Ewa Chabielska, Przemysław Wolak, Katarzyna Głuszek, Justyna Klimek, Piotr M. Zieliński, Michał T. Marzec, Paul B. Savage, Magdalena Parlińska-Wojtan and Robert Bucki

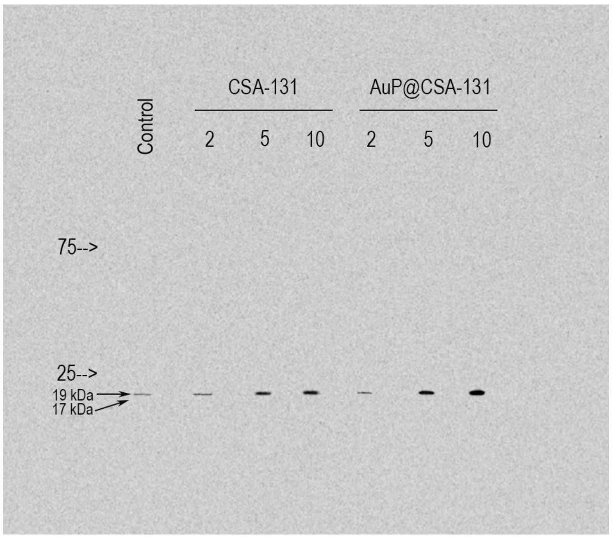

cCasp3 v1.

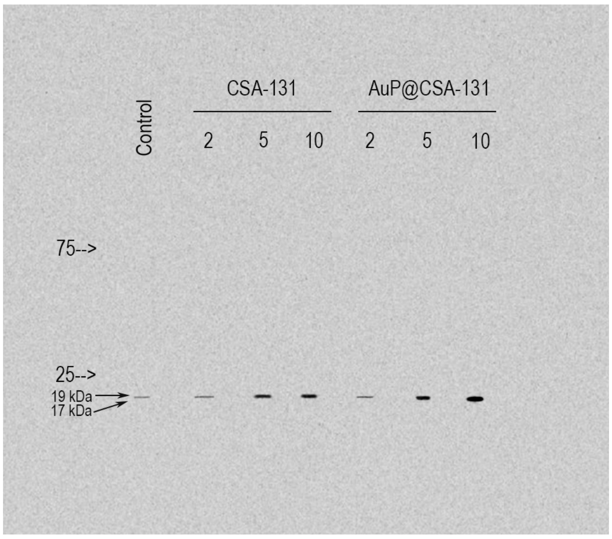

cCasp3 v2.

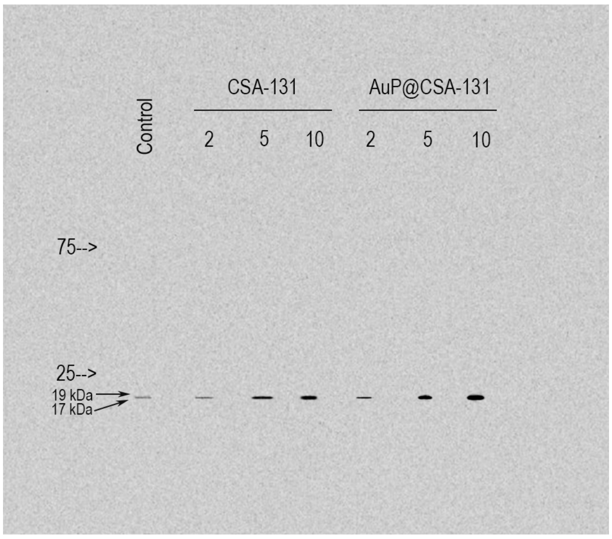

cCasp3 v3_Figure 3B.

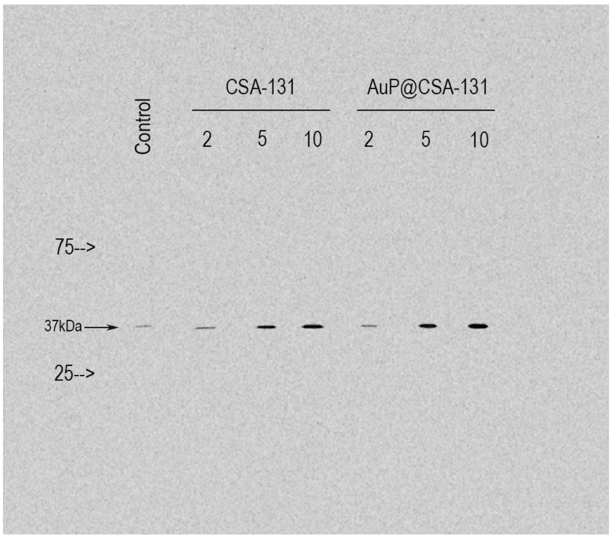

cCasp9 v1.

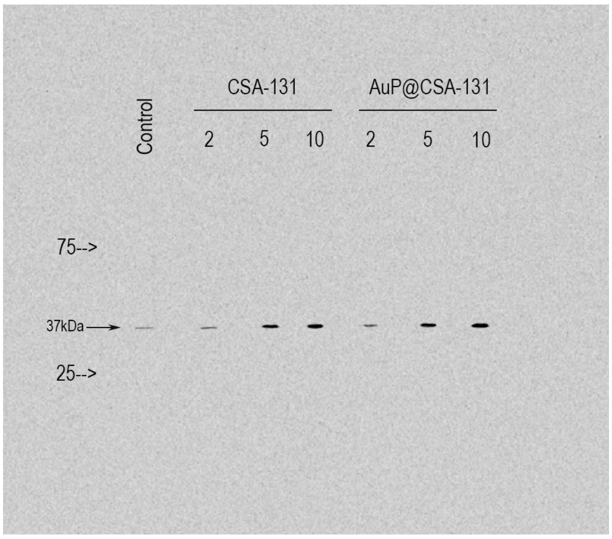

cCasp9 v2.

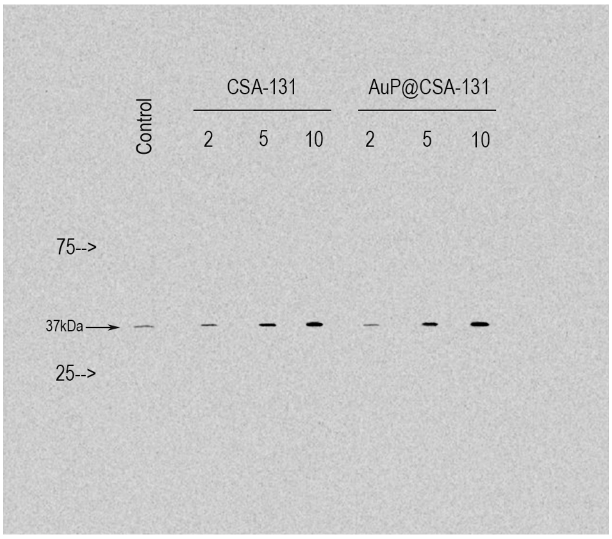

cCasp9 v3_Figure 3B.

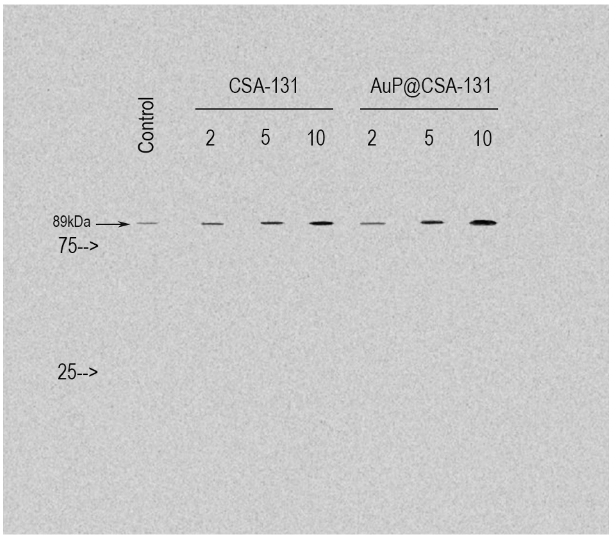

cPARP v1.

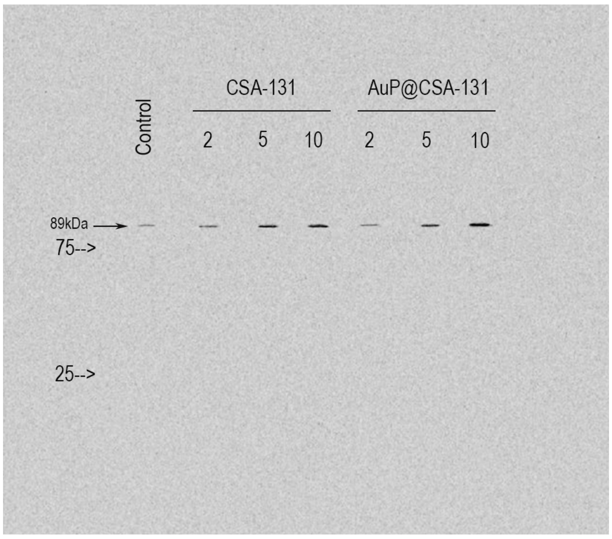

cPARP v2.

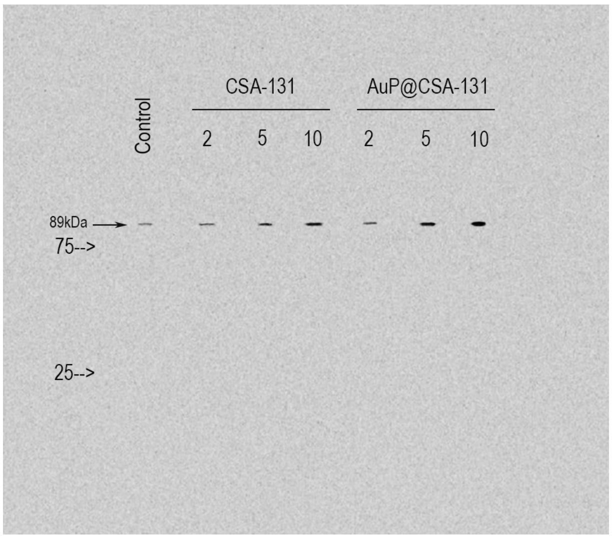

cPARP v3_Figure 3B.

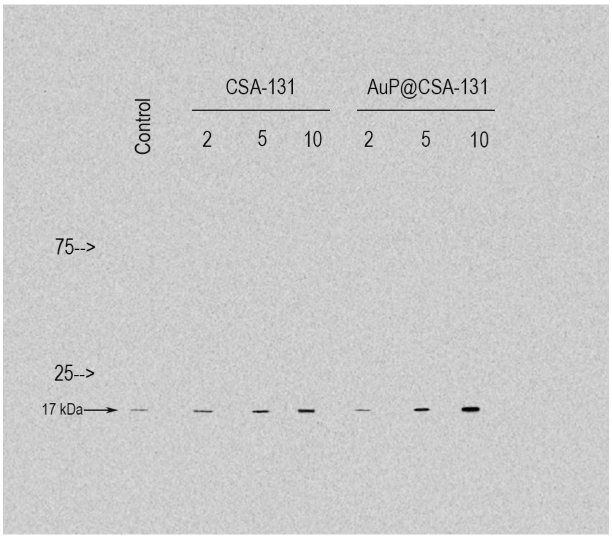

COX v1.

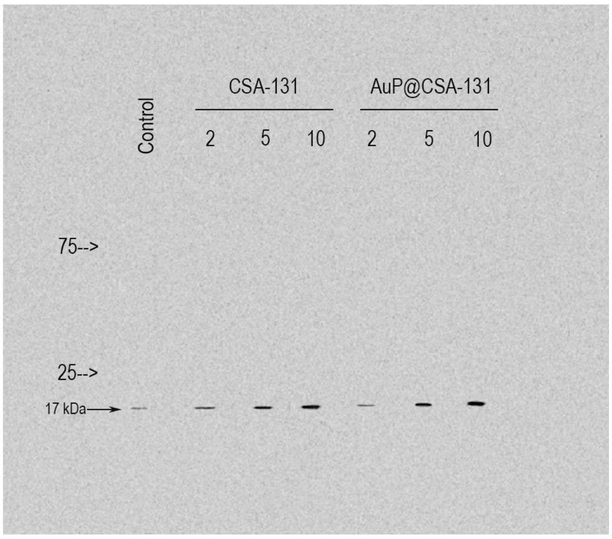

COX v2.

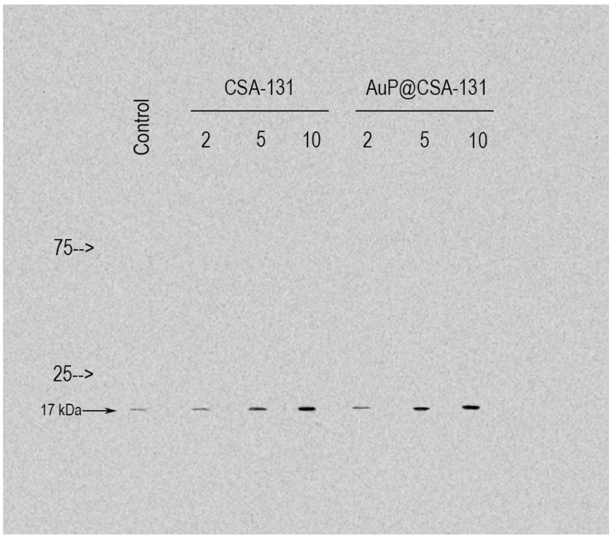

COX v3_Figure 3B.

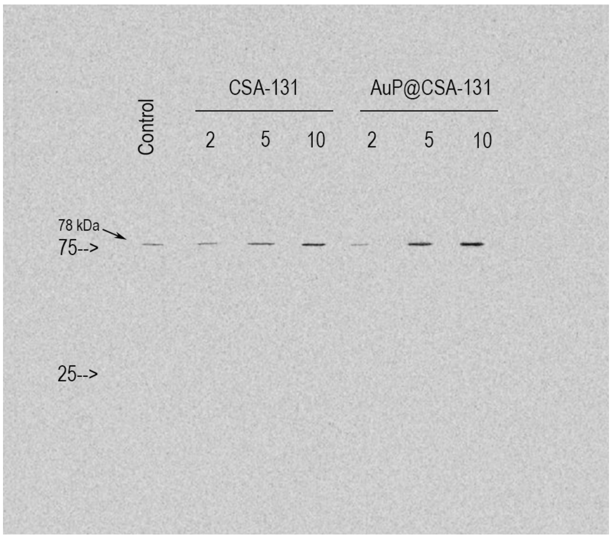

Atg7 v1.

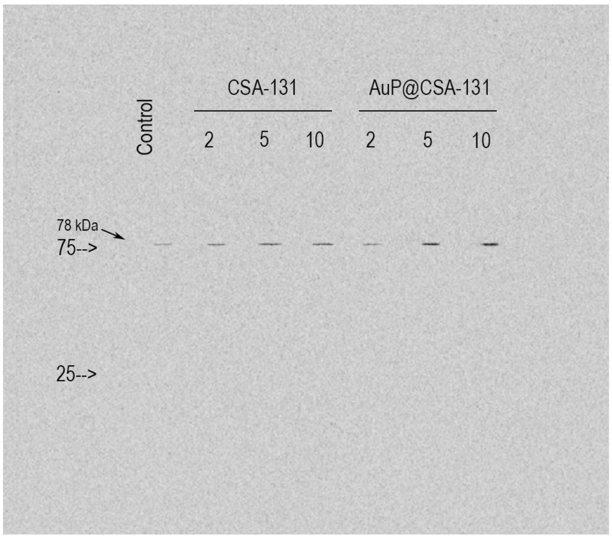

Atg7 v2.

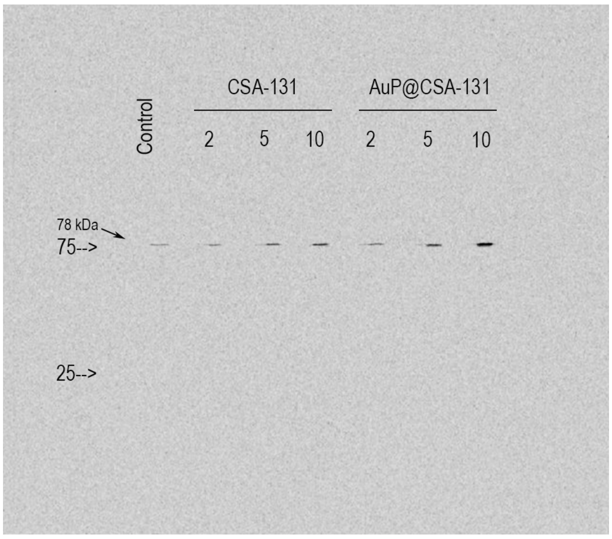

Atg7 v3_Figure 3B.

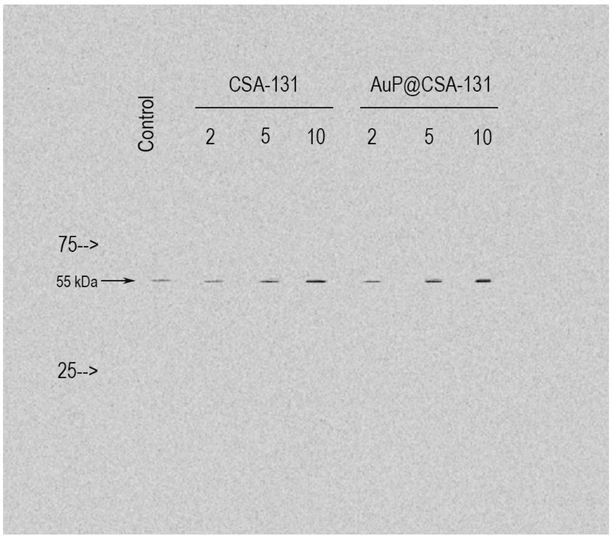

Atg12 v1.

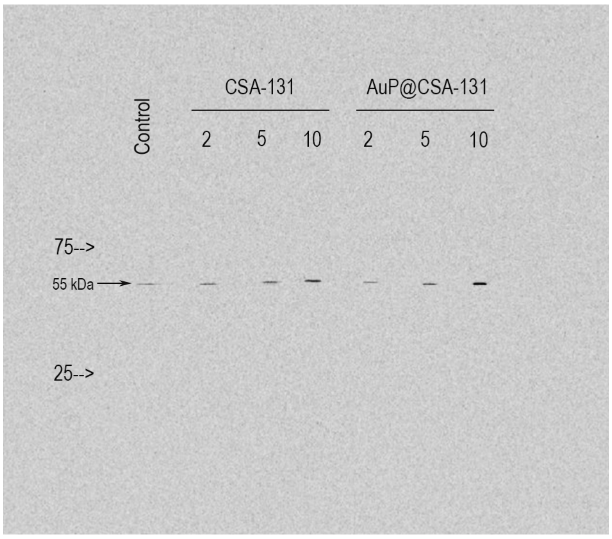

Atg12 v2.

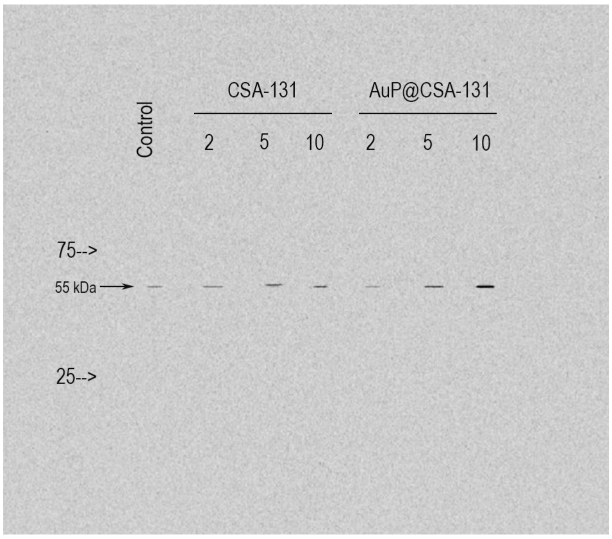

Atg12 v3_Figure 3B.

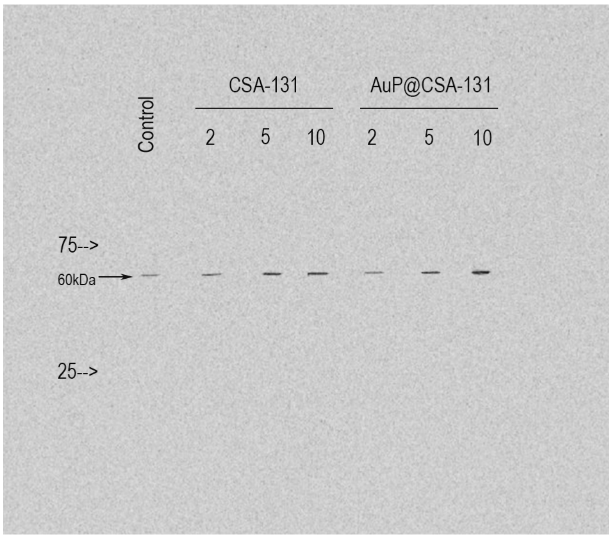

Beclin v1.

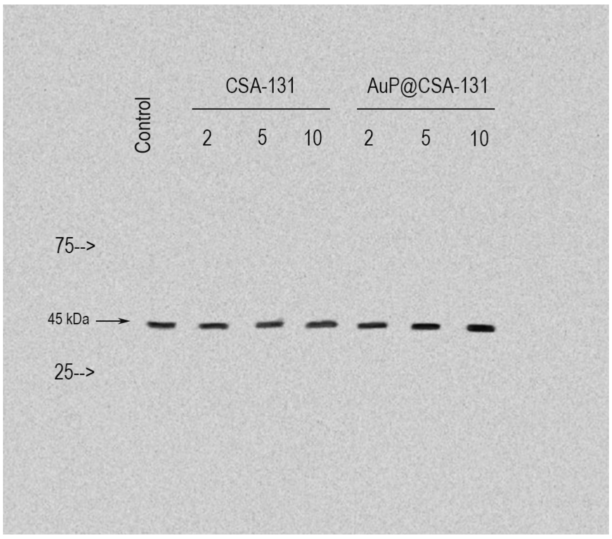

Beclin v2.

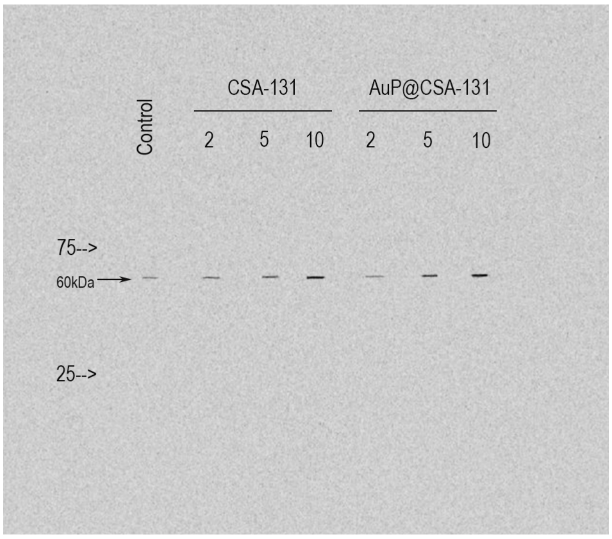

Beclin v3_Figure 3B.

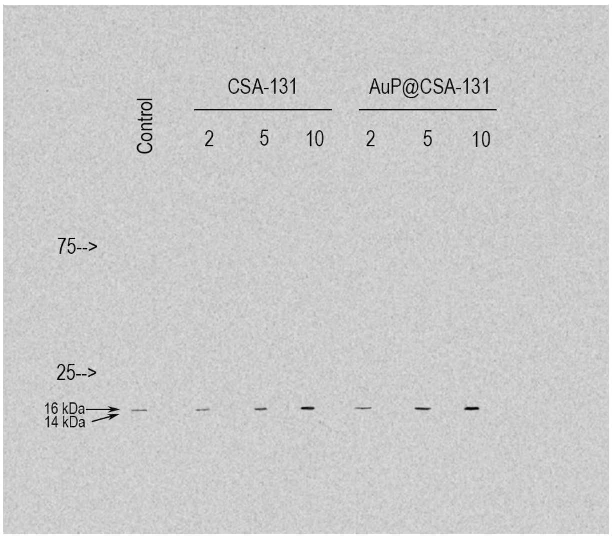

LC3 v1.

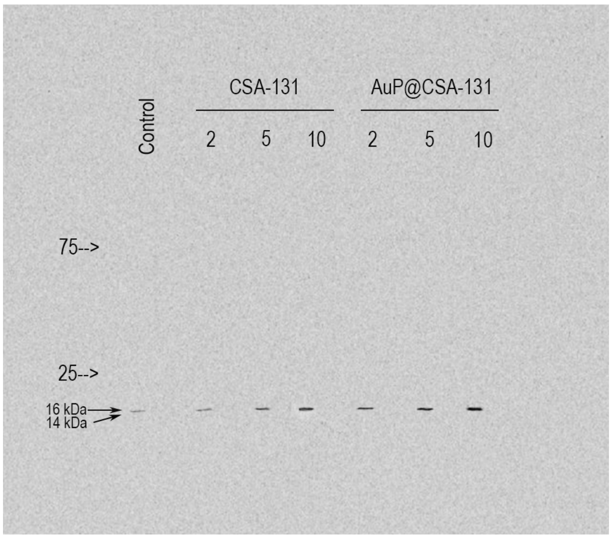

LC3 v2.

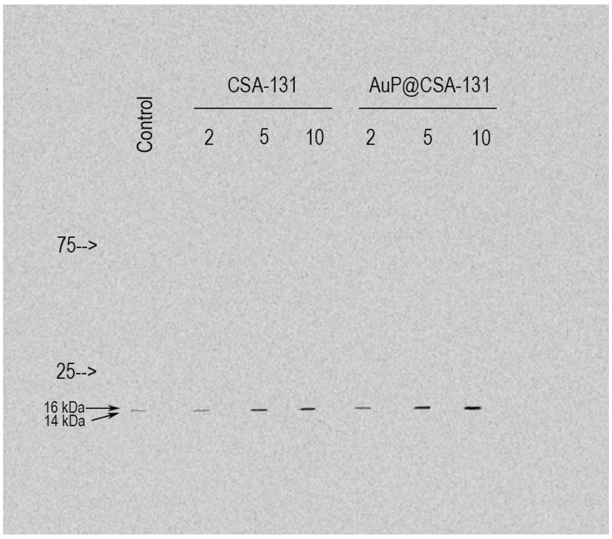

LC3 v3_Figure 3B.

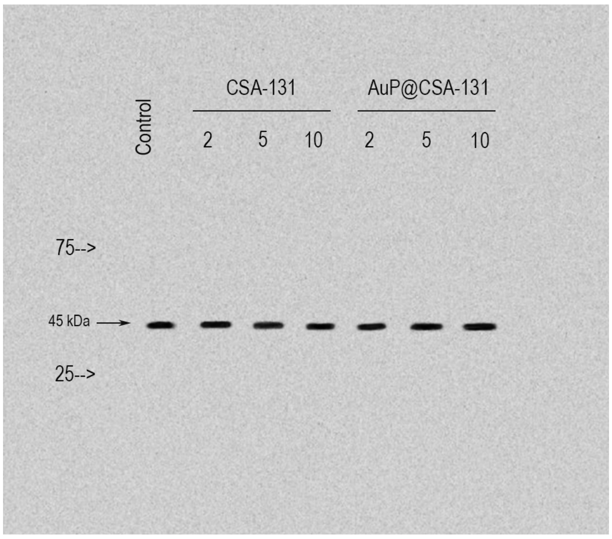

β-actin v1.

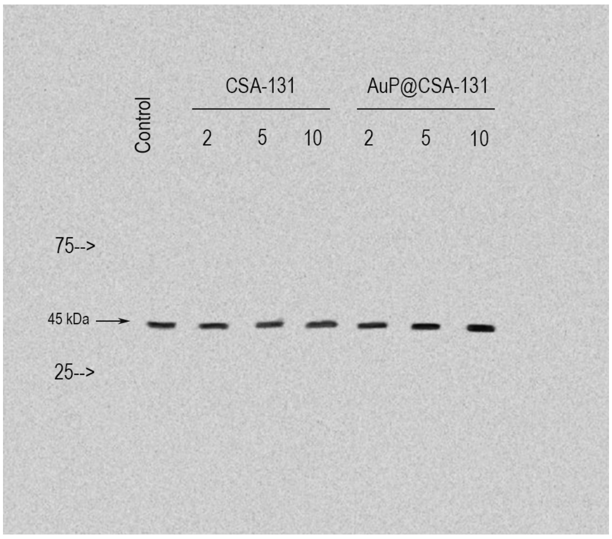

β-actin v2.

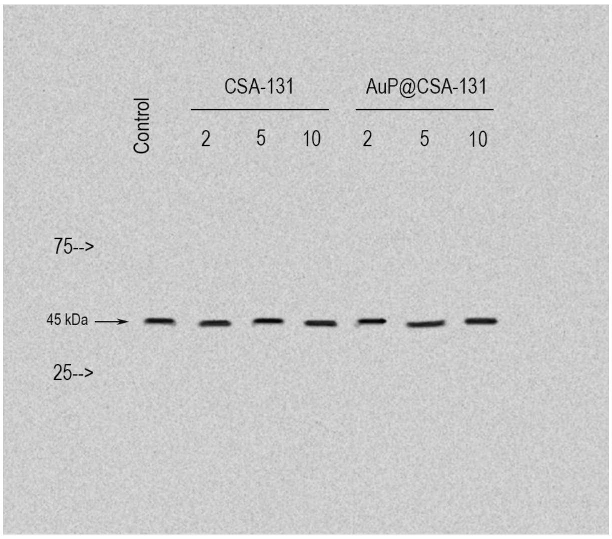

β-actin v3_Figure 3B.

Figure S1. Uncropped Western Blot Figures of Figure 3B.
